# Climate change impact on the potential geographical distribution of two invading Xylosandrus ambrosia beetles

**DOI:** 10.1038/s41598-020-80157-9

**Published:** 2021-01-14

**Authors:** T. Urvois, M. A. Auger-Rozenberg, A. Roques, J. P. Rossi, C. Kerdelhue

**Affiliations:** 1 INRAE, URZF, 45045 Orléans, France; 2 UMR CBGP, INRAE, CIRAD, IRD, Institut Agro, Université Montpellier, Montpellier, France

**Keywords:** Biogeography, Climate-change ecology, Invasive species, Entomology

## Abstract

*Xylosandrus compactus* and *X. crassiusculus* are two polyphagous ambrosia beetles originating from Asia and invasive in circumtropical regions worldwide. Both species were recently reported in Italy and further invaded several other European countries in the following years. We used the MaxEnt algorithm to estimate the suitable areas worldwide for both species under the current climate. We also made future projections for years 2050 and 2070 using 11 different General Circulation Models, for 4 Representative Concentration Pathways (2.6, 4.5, 6.0 and 8.5). Our analyses showed that *X. compactus* has not been reported in all potentially suitable areas yet. Its current distribution in Europe is localised, whereas our results predicted that most of the periphery of the Mediterranean Sea and most of the Atlantic coast of France could be suitable. Outside Europe, our results also predicted Central America, all islands in Southeast Asia and some Oceanian coasts as suitable. Even though our results when modelling its potential distribution under future climates were more variable, the models predicted an increase in suitability poleward and more uncertainty in the circumtropical regions. For *X. crassiusculus*, the same method only yielded poor results, and the models thus could not be used for predictions. We discuss here these results and propose advice about risk prevention and invasion management of both species.

## Introduction

Biological invasions are one of the main threats to biodiversity worldwide, and their magnitude has increased over the last decades with global change^1^. Indeed, climate change alters environmental parameters such as temperature and precipitation patterns and tends to increase the frequency of extreme events^2^. These alterations can decrease ecosystems' resistance and resilience to invasion^3,4^, increase species invasiveness^5^ and initiate range shifts^6^. Human transportation, whether intentional or unintentional, also plays a significant role in shifting species’ range by increasing species passive dispersion^7^, including in normally out-of-reach areas. Moreover, it also increases the propagule pressure (i.e. the number of individuals introduced and the number of introduction events), known to affect establishment probability and ultimately the number of invasive species^8^. Two major challenges of trans-boundary species management are (1) to detect target species at the earliest possible stage and (2) to quickly provide specific actions to regulate or eradicate them^9^. To do so, it is necessary to plan ahead and perform pest risk analyses, which aims at assessing species’ invasion risk considering various biological and environmental characteristics in a given area.

Species distribution modelling (SDM) aims at predicting environment suitability, and thus the potential distribution of a given species in a determined range. SDM is an increasingly important tool for decision-makers when prioritising biodiversity conservation efforts or dealing with invasive species^10^. For the latter, the estimation of habitat suitability can be used to decide how and where to set up detection methods and thus improve early detection probability. SDM uses the relationship between environmental descriptors and the species’ observed distribution to estimate its realised niche^11^, which is used to extrapolate habitat suitability outside of the current species’ range. SDM is also an interesting tool to assess the potential effects of climate change on species distribution^12^. To simulate future climate, scientists rely on Global Circulation Models (GCMs), which attempt to account for all the physical and chemical processes influencing climate, such as ocean, atmosphere, land and sea ice. To predict the effect of greenhouse gases concentration on future climate, each GCM can be run using different radiative forcing scenarios, represented by different Representative Concentration Pathways (RCPs). Four RCPs were used for the IPCC 5th Assessment Report^13^, ranging from the most optimistic one (RCP2.6) to the least optimistic (RCP8.5). Different GCMs are available that all represent a possible future, which we cannot discriminate from. Therefore, GCM selection can potentially affect the results of future distribution modelling^14^, being the primary source of variability between models after the algorithm choice^15^. However, this step is often overlooked and the GCM choice made by default or not justified^12^. When facing biological invasion, it is equally important to determine which areas are suitable and could be threatened, and which ones are consistently predicted as unsuitable. In this regard, using several GCMs allows to explore the uncertainty of SDM predictions and to gain confidence on suitability estimates.

Bark and ambrosia beetles (Coleoptera:Curculionidae:Scolytinae) are considered as one of the most successful groups of invasive species^16^ and represent an increasing concern worldwide^17^. They are hard to detect as they are usually minute insects, and can travel long distances unnoticed, hidden in wood packaging or living plants^18^. Within this group, the species of the genus *Xylosandrus* are especially threatening. Two of them, *X. crassiusculus* and *X. compactus*, native of Southeast Asia, are known invaders worldwide and share similar invasion histories. Both have been detected outside of their native range more than one century ago in Madagascar before spreading to continental Africa and later in the New World. *X. compactus* was detected in North America in 1941 in Florida^19^, in Hawaii in 1964^20^ and in South America in Brazil in 1979^21^. *X. crassiusculus* was first detected in Hawaii in 1950^22^, in North America in South Carolina in 1974^23^ and in South America in Argentina in 2001^24^. Both species were recently reported from Europe where they were first detected in Italy, *X. crassiusculus* in 2003^25^ and *X. compactus* 2011^26^, before being detected in France in 2014 and 2015^27^, respectively. *X. crassiusculus* was then detected in 2016 in Spain^28^ and in 2017 in Slovenia^29^ whilst *X. compactus* was detected in 2019 in Greece^30^ and on the island of Majorca in Spain^31^. As other ambrosia beetles, their ecological characteristics tend to favour invasion. They are xylomycetophagous and feed on symbiotic fungi carried in a specialised structure in their body called mycangium. This feature allows them to attack a broader range of host tree^32^, which is known to be a major reason for their success as invaders^33^. More, siblings mate directly in the maternal gallery, which could allow a single mated female to establish a population. Therefore, they presumably avoid classical detrimental effects of deterministic processes such as mate-finding Allee effect or inbreeding as they are haplodiploid and evolved under inbred mating strategies^34^. Both species originate from subtropical areas and succeeded in invading tropical and subtropical regions in a first step. However, both are now established under temperate and Mediterranean climates, as *X. crassiusculus* is now established as far North as South Canada^35^ and was intercepted several times in the Netherlands during the last decade^36^. We can thus assume that both species are quite plastic, although little is known about their precise ecological requirements.

The first goal of this study was to estimate the potential distribution of both species, with a particular focus on Europe that was recently invaded and where the distribution range of both species is still fairly limited. It is thus now necessary to identify the regions where these invaders still do not occur but could be at risk, to anticipate their expansion and develop preventive management strategies. The second goal of this study is to make predictions about the effect of climate change on both species’ potential distributions in the near future to assess whether some new areas could become suitable or conversely if some area could stay or become unsuitable. To answer both objectives, we performed SDM on *X. crassiusculus* and *X. compactus* using MaxEnt algorithm (1) on current and (2) on future climate models for 2050 and 2070 using 11 GCMs and 4 RCPs. As both species have similar ecological characteristics and a parallel invasion history, we expect that they could threaten the same regions. As hypothesised by Kirkendall and Faccoli^17^, the Mediterranean seems particularly favourable for invasive species with a rich biodiversity and milder winters than the rest of Europe. It corresponds to a biodiversity hotspot that is expected to be particularly susceptible to climate change^37^. Assessing the risk of further invasions in this area is thus of prime importance.

## Material and methods

### Occurrence data and environmental variables

**Figure 1:**
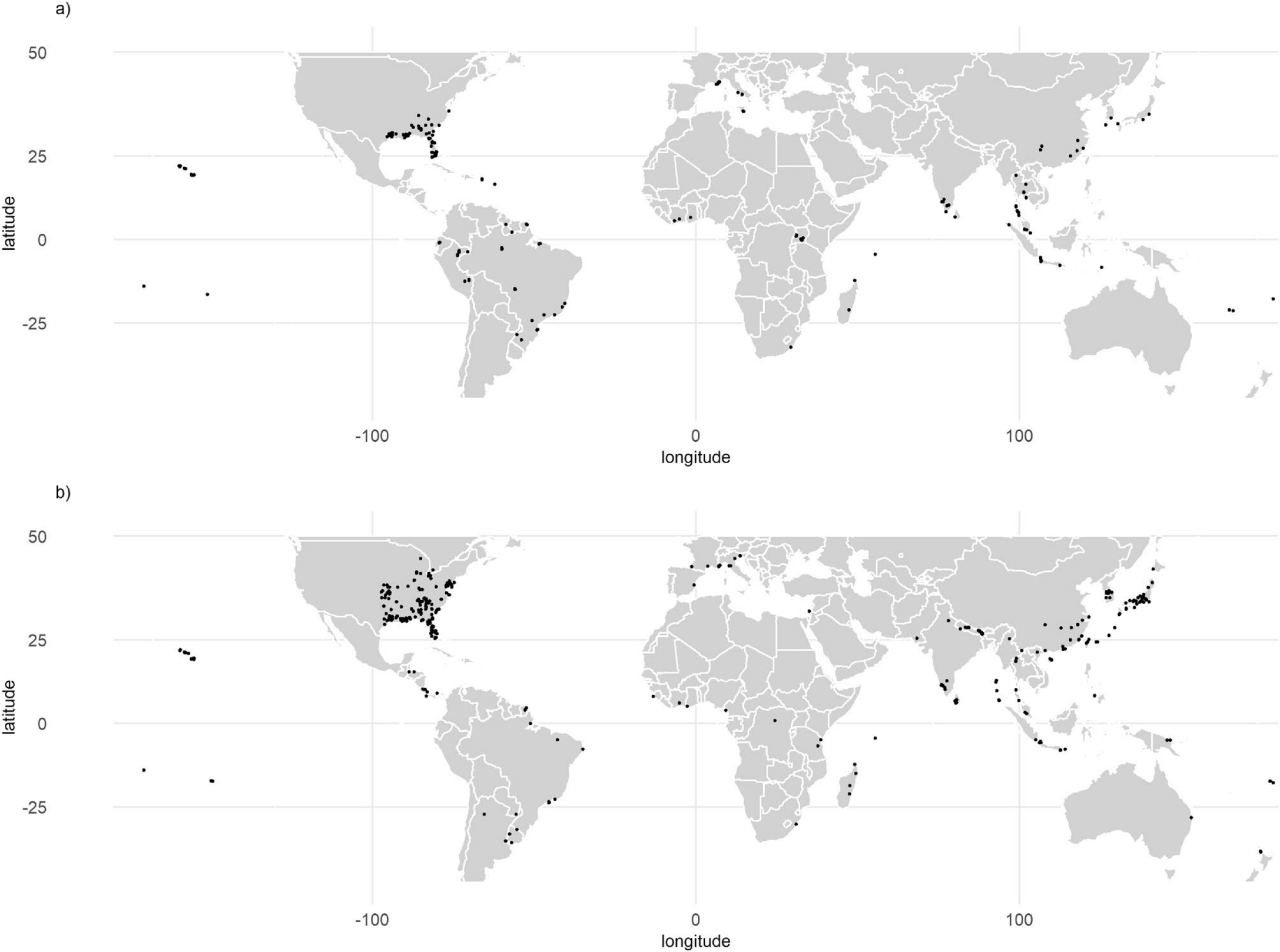
Occurrence data used in the present study of (**a**) *Xylosandrus compactus* (n = 205) and (**b**) *Xylosandrus crassiusculus* (n = 311) after duplicate removal. The maps were generated using R 4.0.0 (https://cran.r-project.org/).

We collected worldwide occurrence records of *X. crassiusculus* and *X. compactus* from the scientific literature (Supplementary Table S1), the Global Biodiversity Information Facility (GBIF) and direct observations up until the 13th of September 2019. Once removed the localities where the species was not proven to be established, we were left with respectively 781 and 323 records for *X. crassiusculus* and *X. compactus*. We then removed duplicated data, by withdrawing all but one occurrence record per pixel of the climate raster (see below), and ended with 311 occurrences for *X. crassiusculus* (33% in its native area) and 205 occurrences for *X. compactus* (30% in its native area) (Fig. 1).

We used environmental variables available from the Worldclim database (version 2.0)^38^, corresponding to the average climate values for 1970–2000, with a resolution of 2.5 arc-min (≈4.5 km at the equator). We selected a priori 11 variables assumed to be potential drivers of *X. crassiusculus* and *X. compactus* distributions. Five were descriptors of the temperature: the temperature seasonality (bio4) and the average temperatures of the wettest, driest, hottest and coldest quarters (respectively bio8, bio9, bio10 and bio11). The remaining 6 variables were the annual precipitations (bio12), the precipitation seasonality (bio15), and the average precipitations of the wettest, driest, hottest and coldest quarters (respectively bio16, bio17, bio18, bio19). Model overfitting is an issue^39^ especially when one aims at using the model to assess future potential distribution^40^. To reduce this issue, we grouped the environmental descriptors by groups of 4 or 5 to produce 126 environmental datasets. Modelling was performed with small sets of variables, hence preventing overfitting.

### Modelling

Because the *Xylosandrus* species under study are invaders in various regions of the world, their spatial range is in constant evolution. In such a non-equilibrium situation, it is very hard to identify locations where the species is absent because environmental conditions are unfavourable (i.e. true absence) rather than because of (transient) dispersal limitations. In such a situation, presence-only algorithms are recommended^10^. We thus used the MaxEnt algorithm^41^, which has been widely adopted in species modelling, notably in biological invasions surveys^42^. To make predictions, MaxEnt relies on presence-only data and background points (i.e. locations where the species’ presence in unknown used as a contrast against presence occurrences).

A first step of the modelling framework consisted in selecting the combinations of climate descriptors that led to the MaxEnt models that best predicted European occurrences when calibrated using occurrences from other regions of the world (see e.g. Godefroid et al.^42^ for an example of such a strategy). To do so, we removed the European occurrences from the datasets of each species, and randomly sampled 80% of the remaining occurrence records to train the model. The remaining 20% of records were used to evaluate the model. To characterise the background environment, 10,000 background points were randomly generated in a wide area representative of the species’ habitat. We did this for 10 replicates for each of the 126 environmental datasets used for each species. We retained a dataset when at least 5 replicates performed (1) an Area Under Curve (AUC) > 0.8, (2) a True Skill Statistics (TSS) > 0.6 and (3) were able to predict 100% of the European occurrences. Such combinations of model × environmental dataset were considered successful and thus used in the next modelling step^43^.

Before running the second step, we searched for the optimal MaxEnt parameters to avoid overfitting^39^ by fitting MaxEnt models with different rate multiplier values (from 0.5 to 4 with increments of 0.5) and six feature classes (1. Linear; 2. Linear + quadratic; 3. hinge; 4. linear + quadratic + hinge; 5. linear + quadratic + hinge + product; 6. linear + quadratic + hinge + product + threshold) using the R package ENMeval^44^. We selected the best combination of MaxEnt parameters based on AIC (see Muscarella et al.^44^ for a full explanation of this method).

In a second step, we used the models and environmental datasets that were selected as explained above with all the occurrence data, thus including the European records. A model was finally kept if it scored an AUC > 0.7. Model outputs were transformed to binary maps using the threshold that optimised the TSS statistics on the testing data^10^. The resulting binary maps were averaged to create a consensus map showing the proportion of models predicting any pixel as suitable^10^.

To visualise the variability of habitat suitability estimates according to the environmental datasets used to calibrate the models, we calculated the standard deviation for each pixel of the consensus maps (Supplementary Fig. S1). We calculated the proportion of area unanimously predicted as respectively suitable and non-suitable, and the proportion of area for which at least 95% of the models agreed upon (i.e. which value is lower than 0.05 or higher than 0.95) which will thereafter be referred to as “high agreement areas”. All these results were computed considering the suitability of landmass (1) worldwide (Supplementary Table S2) and (2) on a restricted focal area including most of Europe and the Mediterranean area, comprised between longitudes 20° W and 50° E and latitudes 27.5  N and 70° N (see Supplementary Table S3).

### Potential distribution under future climate conditions

To assess future distributions, we relied on projections of the environmental variable values based on several GCMs developed by climate institutes^45^. These numerical models can be run using different scenarios of greenhouse gases concentration changes called “Representative Concentration Pathways” (RCPs) and labelled after their radiative forcing value in 2100 (e.g. RCP2.6 or RCP8.5). To predict future distributions, we used future climate projections for 2050 (average for 2041–2060) and 2070 (average for 2061–2080). These estimates were obtained using 11 different GCMs obtained from the WorldClim database (version 1.4) (Table 1), for 4 different RCPs: RCP2.6, RCP4.5, RCP6.0 and RCP8.5. We used the models selected in the second step (see “Results”) to create a total of 12,320 models (combinations of 14 environmental datasets × 10 replicates × 4 RCP × 11 GCMs × 2 years). Models outputs were transformed into binary maps using the threshold that optimised the TSS statistics on the testing data. These binary maps were averaged to create a consensus map per RCP in both 2050 and 2070 that shows for each pixel the projected habitat suitability expressed as a percentage of the models predicting suitable habitat (Supplementary Fig. S2). We also calculated the standard deviation for each pixel of each consensus map (Supplementary Fig. S3).

**Table 1:** Names of the Global Circulation Models used to make future projections and their references.

Code	Global Circulation Model name
*BC*	*BCC-CSM1-1*^74^
*CC*	*CCSM4*^75^
*GS*	*GISS-E2-R*^76^
*HD*	*HadGEM2-AO*^77^
*HE*	*HadGEM2-ES*^77^
*IP*	*IPSL-CM5A-LR*^78^
*MC*	*MIROC5*^79^
*MG*	*MRI-CGCM3*^80^
*MI*	*MIROC-ESM-CHEM*^81^
*MR*	*MIROC-ESM*^81^
*NO*	*NorESM1-M*^82^

Since GCMs differ in some regions and/or for some climate features, they may lead to different model predictions. This variability was expressed as follows (1) for each year we computed the average habitat suitability over the 11 GCMs for a given RCP (Supplementary Fig. S4), (2) this average was used to centre projections from each GCM. The resulting maps (one by GCM) (Supplementary Fig. S5) finally displayed a negative value when the GCM considered predicted a lower suitability than the average of all GCMs and a positive value otherwise.

We used the R software^46^ and the following R packages to perform modelling and render the maps: dismo^47^, biomod2^48^, ENMeval^44^, cowplot^49^, ggplot2^50^, rnaturalearth^51^ and raster^52^.

## Results

### SDM under current conditions

**Figure 2:**
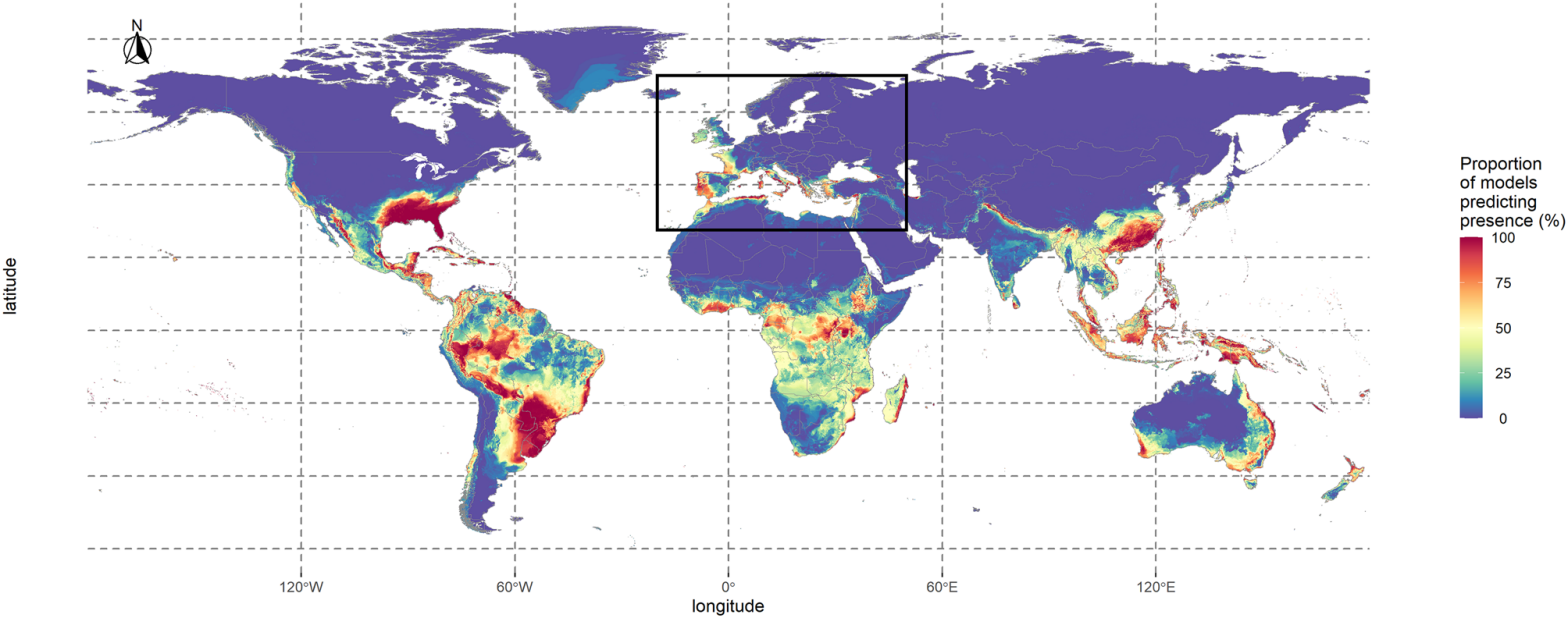
Consensus map showing habitat suitability for *Xylosandrus compactus* under current climate conditions. This consensus map was computed by averaging binary maps and represents the percentage of models predicting each pixel as suitable. The black square represents the limits of the focal area. The map was generated using R 4.0.0 (https://cran.r-project.org/).

Out of the 126 environmental datasets tested in the first step of the analysis, none fulfilled the evaluation criteria for *Xylosandrus crassiusculus*, either due to lack of predictive power regarding European occurrences, low AUC (< 0.8) or low TSS (< 0.6) values. Consequently, we could not perform ecological niche modelling on this species, and the results presented hereafter only concern *X. compactus*. For this species, 14 datasets fulfilled the evaluation criteria of step 1 and were thus used in step 2. Supplementary Table S4 shows a summary of the evaluation metrics for the models retained to perform the consensus map (Fig. 2). Under the current climate, 55% of the world surface was unanimously predicted as non-suitable, whereas 1.13% was unanimously predicted as suitable. In the focal area, these predictions were respectively 66% and 0.23%. There was a high agreement for 65% of the world; this number reached 77% in the focal area, where at least 25% of the models predicted most Mediterranean coasts and islands as suitable, except for Tunisia, Libya, Egypt and Southeastern Spain. The models also predicted the Western coasts of Spain, France, up to the UK as suitable, although the values decreased northwards. In most places, the suitability decreased with distance from the coast. Outside of the focal area, a substantial part of the models predicted Central America, all islands in Southeast Asia, Nepal, and some Australian coasts as suitable.

### Modelling distributions under future climate estimates

**Figure 3:**
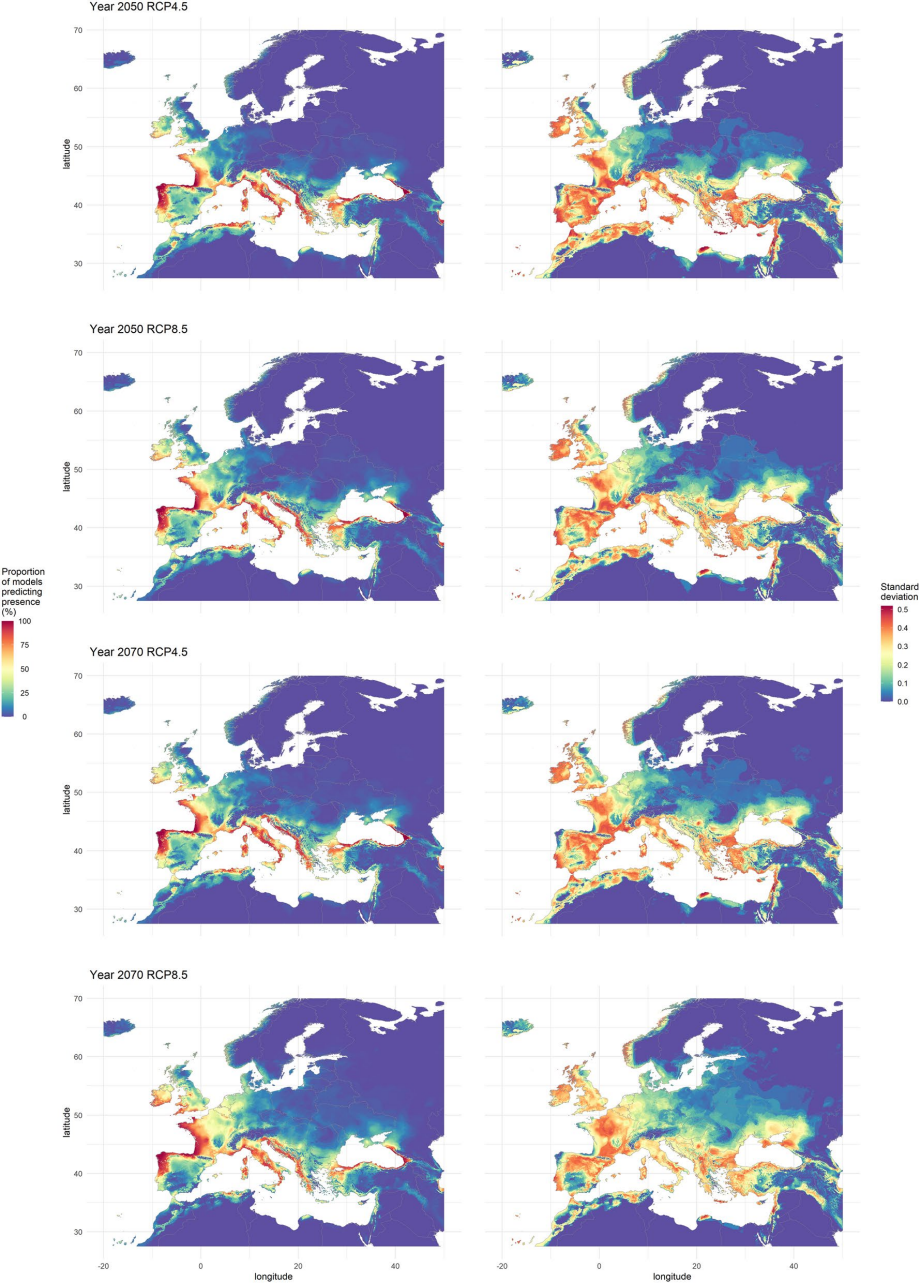
On the left side, consensus maps showing habitat suitability for *Xylosandrus compactus* under future climate for 2 greenhouse gases concentration scenarios (RCP4.5 and 8.5) and for 2 years (2050 and 2070). These consensus maps were computed by averaging presence–absence maps and represent the percentage of models predicting each pixel as suitable. On the right side, maps representing the standard deviation of their left counterpart where the higher values the lower the agreement between models’ predictions. The maps were generated using R 4.0.0 (https://cran.r-project.org/).

In both 2050 and 2070 and for all RCPs, ca. 50% of the world was unanimously predicted as non-suitable (Supplementary Fig. S2). Conversely, less than 0.35% was consensually predicted as suitable, ranging from 0.19% for the RCP2.6 in 2050 to 0.31% for the RCP8.5 in 2070. In the focal area, 55% of the land territory was unanimously predicted as non-suitable and around 0.1% was consensually predicted as suitable (Fig. 3). Yet, the results for 2070 with RCP8.5 in Europe stood out with 45% of the world unanimously predicted as non-suitable and only 0.02% of the area unanimously predicted as suitable. Around 60% of the world and 70% of the focal area reached a high agreement. For the 4 RCPs, we observed a progression of the habitat suitability towards North and Northeast between the current climate and the projections for 2050 in the focal area, where the consensus map shows that the majority of Western Europe would become suitable. However, the suitability was predicted to decrease in Southwestern Spain, along the coasts of Morocco and Algeria, and in the easternmost parts of the Mediterranean (Israel, Turkey, Greece). We observed the same trends between 2050 and 2070 for the RCP4.5, 6.0 and 8.5. Over the same period, we observed the opposite trend for the RCP2.6, with a regression of the habitat suitability in the North and Northeastern areas and a progression in North Africa and East of the Mediterranean Sea. Outside the focal area, we observed an increase of the habitat suitability between 2050 and 2070 in Northern USA, around Uruguay, in central Africa, in Oceania and some regions of Asia. Conversely, we observed a regression around Venezuela, Central America and some areas in the USA.

The standard deviation of predicted habitat suitability is given in Fig. 3 and in Supplementary material, Fig. S3. These maps highlight the areas where models’ projections diverge, showing low values near the coastline, where most models predict on high suitability, and in the North-east Europe, where most models agree predict low suitability.

**Figure 4:**
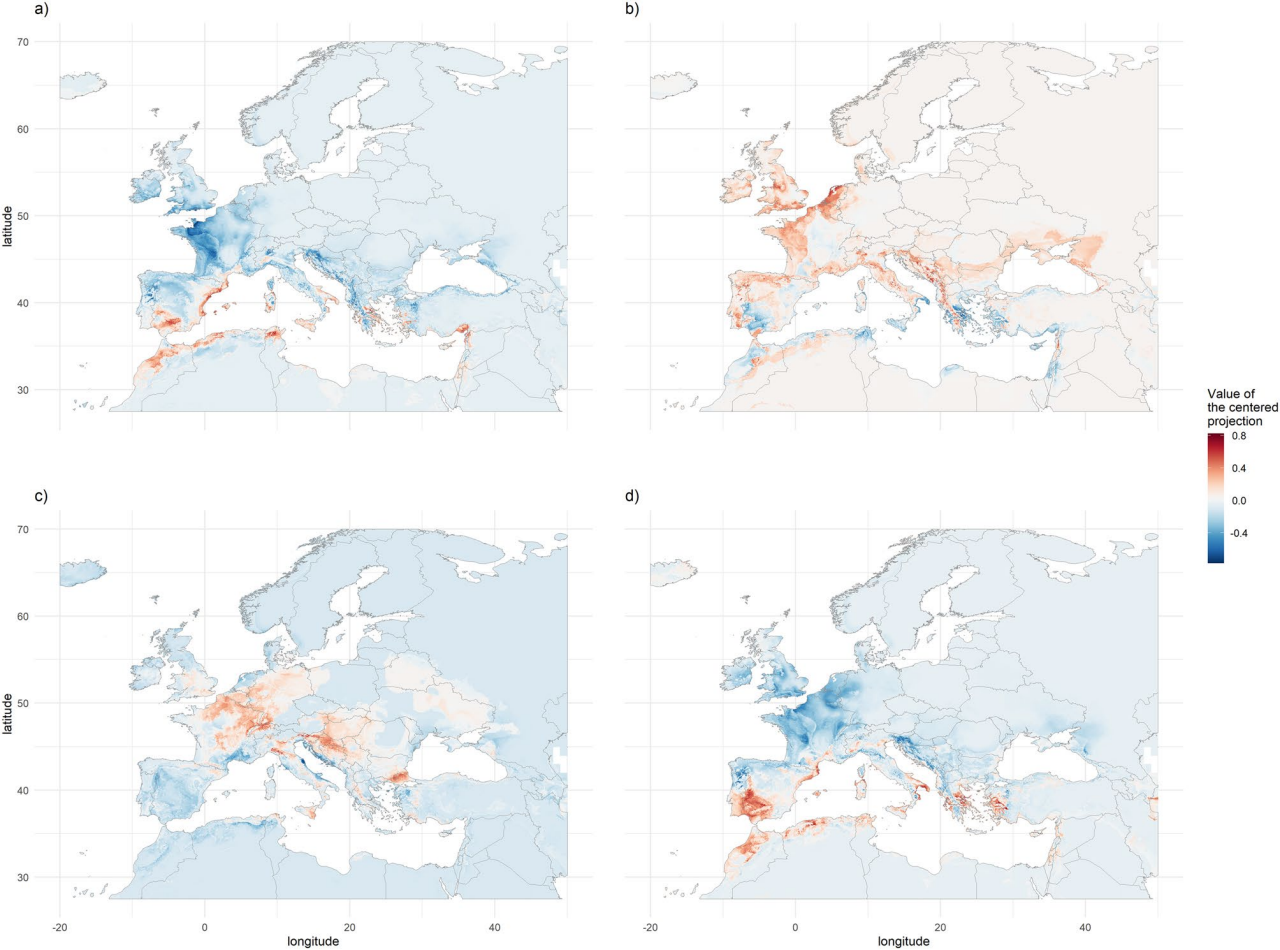
Illustration of the variability between the GCMs used to create consensus maps using centered and standardised maps. These display a negative value when the GCM considered predicts a lower suitability than the average of all GCMs and a positive value otherwise. A and B correspond to two GCMs (MG and MI respectively) used to compute the RCP2.6 maps for the year 2050, C and D represent two GCM (HD and MG respectively) used to compute the RCP8.5 for the year 2070. The maps were generated using R 4.0.0 (https://cran.r-project.org/).

We observed the same type of predictions when comparing the GCM maps between 2050 and 2070 for each RCP. This is also true for most comparisons between RCPs amongst each predicted year. However, this was not true when comparing the predictions between GCMs, which generated divergent results. Figure 4 highlights the variability of the model outputs according to GCMs for a given scenario. While maps A and D both displayed under average suitability in Western Europe and the Balkans and over average values near Gibraltar, maps B and C displayed symmetrical results. On the same principle, we were able to group GCMs according to their predictions. For the year 2050 and RCP2.6, the maps for the GCMs HD and HE displayed above-average suitability over the focal area. Conversely, the GCMs CC, GS and MG predicted a lower than average suitability for most of the focal area but South-eastern Spain and Northern Africa. The other GCMs patterns were less structured (IP, MI and MR) or close to the average predictions (BC, NO, MC). At the worldwide scale, NO and MG predicted above-average suitability in Eastern China, South-eastern USA and Northern South America until Paraguay and North America. On the contrary, HE, MI and MR predicted below average suitability for these regions.

## Discussion

Several pest risk analyses have been performed on species of the *Xylosandrus* genus in Europe in the last decade (UK^53^, France^54^, Slovenia^55^). However, while they assessed the species’ ability to enter, establish and spread in the area of interest, the establishment part mostly focused on the presence of host species and lacked information about climate suitability. Our study provides the first attempt to model the potential distribution of *Xylosandrus compactus* and *X. crassiusculus*, two invasive species whose range has been increasing ceaselessly during the last century. Both species have similar ecology and invasion history, suggesting that their potential distribution could be quite similar and thus that their respective models would produce analogous outputs. However, surprisingly, no model created for *X. crassiusculus* matched our criteria.

### Failure to model *Xylosandrus crassiusculus*’ species distribution

The failure in appropriately modelling *X. crassiusculus*’ potential distribution could be explained by several factors, mutually compatible.

Our first hypothesis is that we may have overlooked crucial information when performing SDM on *X. crassiusculus*. According to the literature, *X. crassiusculus* is genetically structured and could be divided into two or more differentiated clades, corresponding to potential cryptic diversity^56,57^. It has been shown that integrating phylogeographical information into SDM can alter the results when performing SDM on different clades of a species^58,59^. Yet, as genetic studies concerning *X. crassiusculus* in different regions of the world did not rely on similar molecular markers, it is not possible at present to determine which clade occurs in a given region. We thus tested the SDM approach at the species level, i.e. all occurrences were used to calibrate the model. In doing so, we possibly grouped clades with potentially different ecological features, hence building models with weak predictive power. To counter this problem, we are currently working on a comprehensive genetic analysis of *X. crassiusculus* worldwide, to get a better insight about its genetic structure and assign a clade to each locality. This would then allow us to perform a SDM for each clade separately.

Another hypothesis is that SDM relies on several assumptions that can easily be violated when working on expanding invasive species. One is that species are supposed to be at equilibrium, which means that they should be present in all suitable areas^60^. However, we know that *X. crassiusculus* is still expanding. Moreover, even though we reached more than 300 occurrence records, we might have under-sampled the native area, which could prevent us from inferring *X. crassiusculus*’ realised niche. Indeed, particular populations can sometimes evolve to face a harsher environment, and thus be preadapted to certain conditions, which ease further invasion^61^. Failing to include such peculiar populations might affect SDM outputs, although it is difficult to evaluate the magnitude of such effect. A last possible explanation for SDM failure could be related to variable selection, but this latter possibility appears very unlikely as we followed the same procedure as for *X. compactus* and created models with 126 variable datasets made of 11 environmental variables.

### Potential distribution of *X. compactus* under current climate

Correlative SDM uses relationships between environmental predictors and species’ occurrences to predict its potential distribution. Therefore, unreliable information regarding *X. compactus*’ distribution such as countries for which exact occurrence data were not mentioned, or localities where the species was detected but was not proved to have settled, had to be removed from datasets. Despite these precautions, our model successfully spotted suitable areas that were not initially included in the occurrence dataset (e.g., in Central Africa and Oceania) for *X. compactus*. Similarly, the models predicted the Greek province of Peloponnese and the Balearic Island Mallorca, where it was reported as established after we stopped gathering occurrence data^30,31^, which suggests that the models have good predictive power. According to our model, *X. compactus*’ distribution in America could expand to Chile and Argentina, all Central America and the Western coast of North America. In the focal area, most of the Mediterranean coasts and the westernmost parts of the UK are predicted as suitable. Besides Northwest Australia, almost all Oceania and South East Asia is expected to be suitable. This suggests that even in its native area in Asia, *X. compactus* might colonise new areas or islands where it is not present yet. Even though *X. compactus* has proven to be very adaptable, invading different climatic regions in the last century, our models unanimously predicted more than 50% of the world’s area as not suitable. Most of these areas are considered arid or semi-arid^62^ whether hot (e.g. Sahara or Arabian Peninsula) or cold (e.g. Canada or Andean Mountains). Little is known about the critical thermal minimum and maximum limits in species of the *Xylosandrus* genus, which could be used as thresholds constraining *X. compactus*’ distribution. Gugliozzo et al.^63^ fitted a linear relationship between mortality and minimum temperature and found a 40% mortality in overwintering adults when the minimum temperatures decreased to 5 °C. The study did not record temperature under 5 °C, far from the lower lethal temperature of 10 °C observed for *Xyleborus glabratus*, another ambrosia beetle^64^. Some *Xylosandrus* species, including *X. compactus*, showed an absence of flight activity under 20 °C^65,66^, a temperature threshold which may act as another constraining parameter. *X. compactus*’ distribution is expected to shift with climate change as precipitations and temperature patterns are modified.

### Potential distribution of *X. compactus* under future climate conditions

The direction and magnitude of a range shift depend on how climate change will affect the environmental parameters that constrain the species’ distribution^67^. Being generalist and carrying its symbiotic fungi to feed on, *X. compactus*’ distribution is expected to be mostly dependent on temperature and humidity. However, *X. compactus* lives most of its life in galleries, where wood acts as a buffer, protecting individuals from ambient air temperature and extreme events^64^. The uncertainties of predictions were higher when dealing with future conditions compared to the consensus obtained under the current climate. Indeed, apart from Central Africa, the values observed on the consensus maps are usually lower. Whereas the areas predicted as suitable by at least some models stretches polewards in the Northern hemisphere, the predicted suitability in the Southern hemisphere is less consistent, decreasing over time in some places but not in others. In the focal area, the habitat suitability is predicted to increase going North and Northeast, reaching Central Europe, the Balkans and the Black Sea. However, outside the focal area, no new country is projected to become suitable between now and 2050 or 2070.

Even though some GCMs can make close predictions for the environmental parameters’ value in the future, it is necessary to use several of them when predicting species’ potential distribution. Indeed, when using general-purpose machine learning methods such as MaxEnt, which relies on complex relations between the predictors to assess if areas are suitable or not, even small differences could lead to unpredictable and substantial differences in the final predictions^12^. Moreover, performing models over a range of GCMs and RCPs provide decision-makers more reliable information regarding species’ potential distribution under future climate, notably regarding the areas predicted as unsuitable. On this point, our study is remarkable as it uses an exceptionally high number of GCMs, sometimes predicting divergent if not symmetrical results, and 4 RCPs, aimed at representing four different potential futures. Indeed, in their review, Porfirio et al.^12^ found that 40% of the studies (out of 163) used 2 or more GCMs, with only 7 using more than 10 GCMs, all being focused on methodology and not on conservation issues.

### Risk prevention and invasion management

In addition to altering ambrosia beetles’ distribution, climate change might increase the damage they cause^68^. On the one hand, temperature changes could facilitate their survival and development and thus significantly modify their population dynamics. On the other hand, changes in precipitations pattern would impact tree by prompting stress-induced ethanol emissions, which might increase their susceptibility to ambrosia beetles^69,70^. While ambrosia beetles have been eradicated when established in very localised areas^71^, this strategy is most of the time unsuccessful. Indeed, they live most of their lives inside galleries where they are protected from pesticides, parasitoids and predators. Moreover, these ecological characteristics make them difficult to detect, which prevent efficient eradication strategies. Even if cutting and destroying infested trees can reduce population density, it is not applicable with high population densities or already widespread species.

The most cost effective management strategy is to prevent species’ invasion in the first place. Wood transportation is a major introduction pathway for bark and ambrosia beetles^68^, allowing them to disperse over long distances passively. However, even if phytosanitary measures are in place to prevent involuntarily transporting species (e.g. heat treatment or fumigation of the wood), they are not sufficient to ensure that no specimen reaches its destination alive. Moreover, bark and ambrosia beetles are also known to travel in living plants, which do not comply with the same regulations. To limit species’ invasion through wood and living plant transportation, a strengthening of the regulation might be necessary. However, resources are limited, so it is essential to prioritise areas to survey. For monophagous or oligophagous invasive species, the invasion pathways and currently suitable areas can be determined using host species transportation and distribution. However, both *X. compactus* and *X. crassiusculus* are known to have a broad range of hosts^31,72^, and more are added to the list as they invade new regions^73^. Hence, managers should not rely on pre-existing host lists as a way to consider an area as unsuitable. Our result relies on environmental parameters to show which areas are suitable for *X. compactus*. This could significantly improve the future pest risk analyses, in addition to being a helpful tool for decision-makers when making policies about trapping for early detection of *X. compactus*. Indeed, our results show that some areas are still free of *X. compactus* even though they are predicted as suitable, today or in the future. We suggest that such areas should be prioritised for early detection strategy, while efforts could be partially relaxed in regions unanimously predicted as unsuitable in the present study.

## Supplementary Information

Supplementary Information 1.

Supplementary Information 2.

Supplementary Information 3.

Supplementary Information 4.

Supplementary Information 5.

Supplementary Information 6.

Supplementary Information 7.

Supplementary Information 8.

Supplementary Information 9.

Supplementary Information 10.

Supplementary Information 11.

Supplementary Information 12.

Supplementary Information 13.

Supplementary Information 14.

Supplementary Information 15.

Supplementary Information 16.

Supplementary Information 17.

Supplementary Information 18.

Supplementary Information 19.

Supplementary Information 20.

Supplementary Information 21.

Supplementary Information 22.

Supplementary Information 23.
