# Supplementary material for: Climate change impact on the potential geographical distribution of two invading Xylosandrus ambrosia beetles
Source: Sci Rep. 2021 Jan 14;11:1339. doi: 10.1038/s41598-020-80157-9 (PMC7809213; doi:10.1038/s41598-020-80157-9)
Supplement: Supplementary file 1 — Supplementary Information 1. [file 41598_2020_80157_MOESM1_ESM.pdf]

Climate change impact on the potential geographical distribution of two invading *Xylosandrus* ambrosia beetles

T. Urvois, M.A. Auger-Rozenberg, A. Roques, J.P. Rossi, C. Kerdelhue

Figure S1: Map showing the standard deviation of the habitat suitability worldwide for *Xylosandrus compactus* under current climate conditions. The higher values the lower the agreement between models' predictions. This map was created using R 4.0.0 (https://cran.r-project.org/) by computing the standard deviation for each pixel.

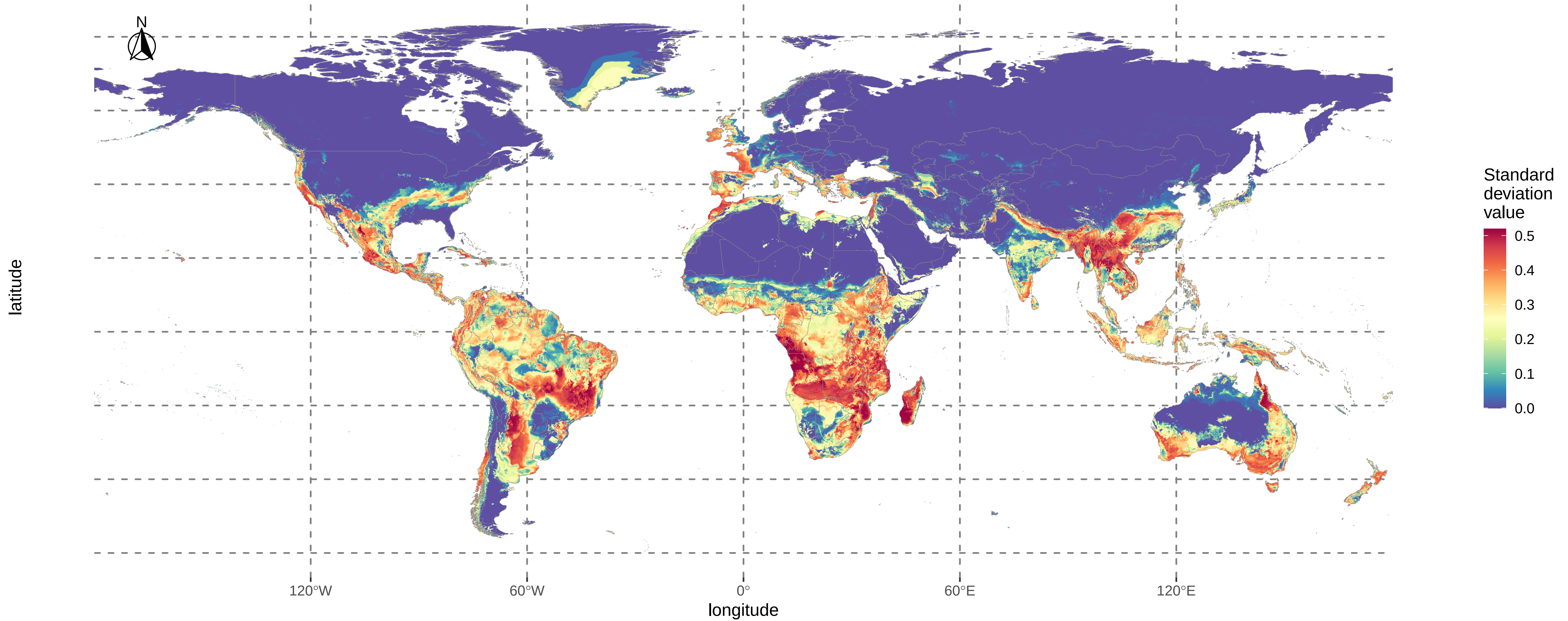

Figure S1: Map showing the standard deviation of the habitat suitability for Xylosandrus compactus under current climate conditions. This map was created by computing the standard deviation for each pixel.
