# Supplementary material for: Climate change impact on the potential geographical distribution of two invading Xylosandrus ambrosia beetles
Source: Sci Rep. 2021 Jan 14;11:1339. doi: 10.1038/s41598-020-80157-9 (PMC7809213; doi:10.1038/s41598-020-80157-9)
Supplement: Supplementary file 2 — Supplementary Information 2. [file 41598_2020_80157_MOESM2_ESM.pdf]

Climate change impact on the potential geographical distribution of two invading *Xylosandrus* ambrosia beetles

T. Urvois, M.A. Auger-Rozenberg, A. Roques, J.P. Rossi, C. Kerdelhue

Figure S2: Consensus maps showing the habitat suitability worldwide for *Xylosandrus compactus* in 2050 and 2070 according to the RCPs 2.6, 4.5, 6.0 and 8.5. These consensus maps were computed by averaging presence-absence maps and represent the percentage of models predicting each pixel as suitable. The maps were generated using R 4.0.0 (https://cran.r-project.org/).

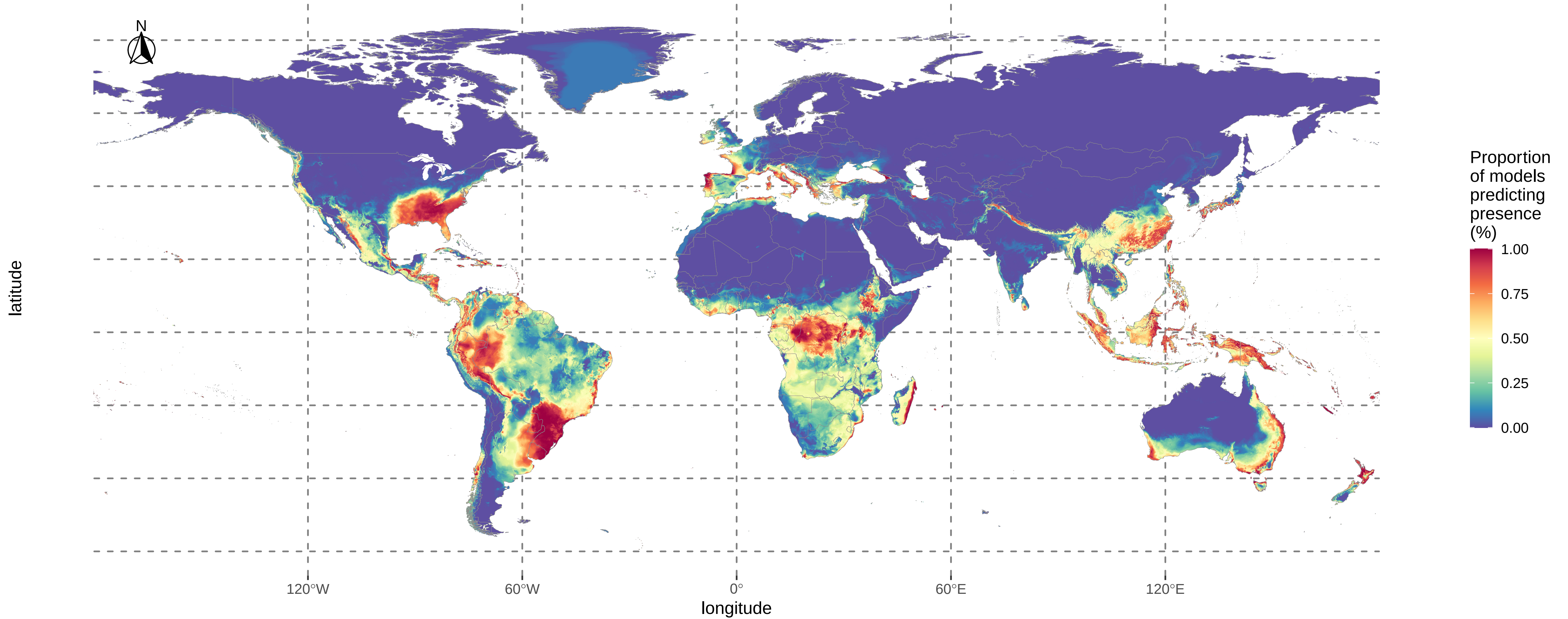

Figure S2−A: Consensus map showing the habitat suitability worldwide for Xylosandrus compactus in 2050 according to the RCP2.6.
Hot colours represent areas where the models reached a good agreement.

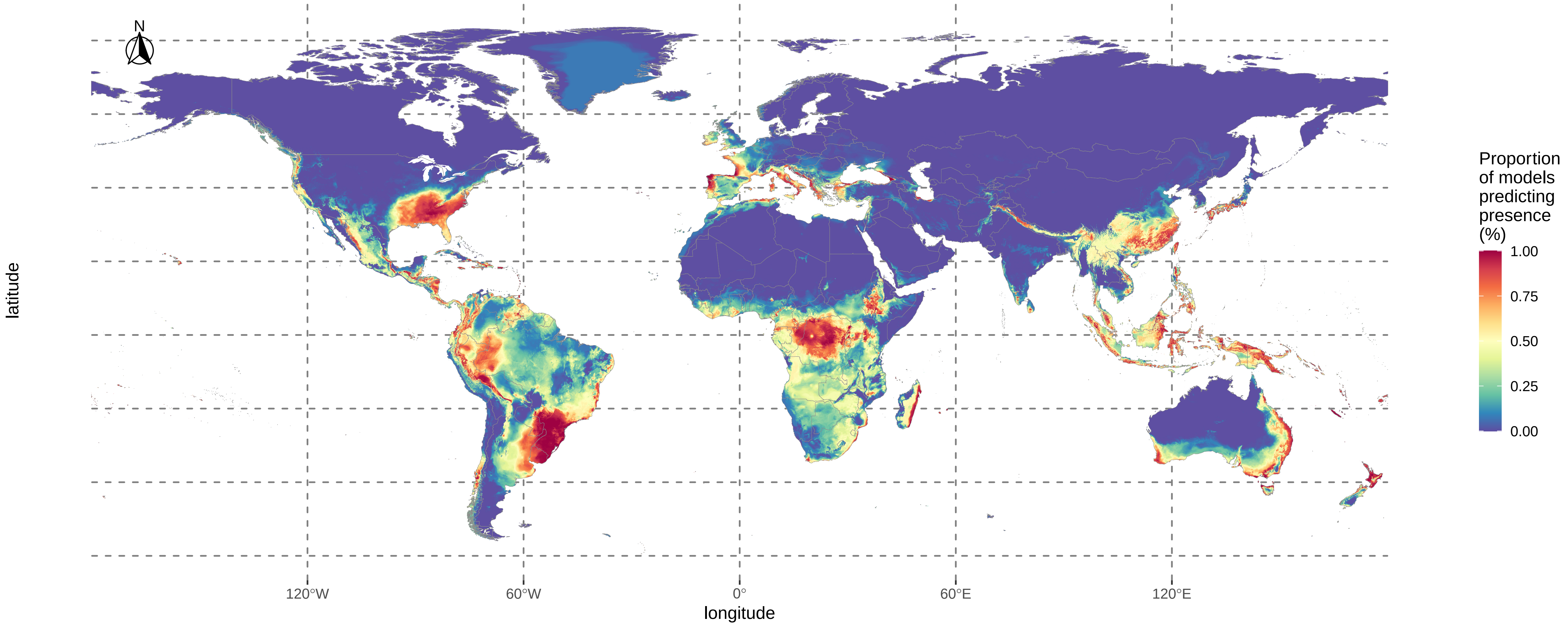

Figure S2−B: Consensus map showing the habitat suitability worldwide for Xylosandrus compactus in 2050 according to the RCP4.5.
Hot colours represent areas where the models reached a good agreement.

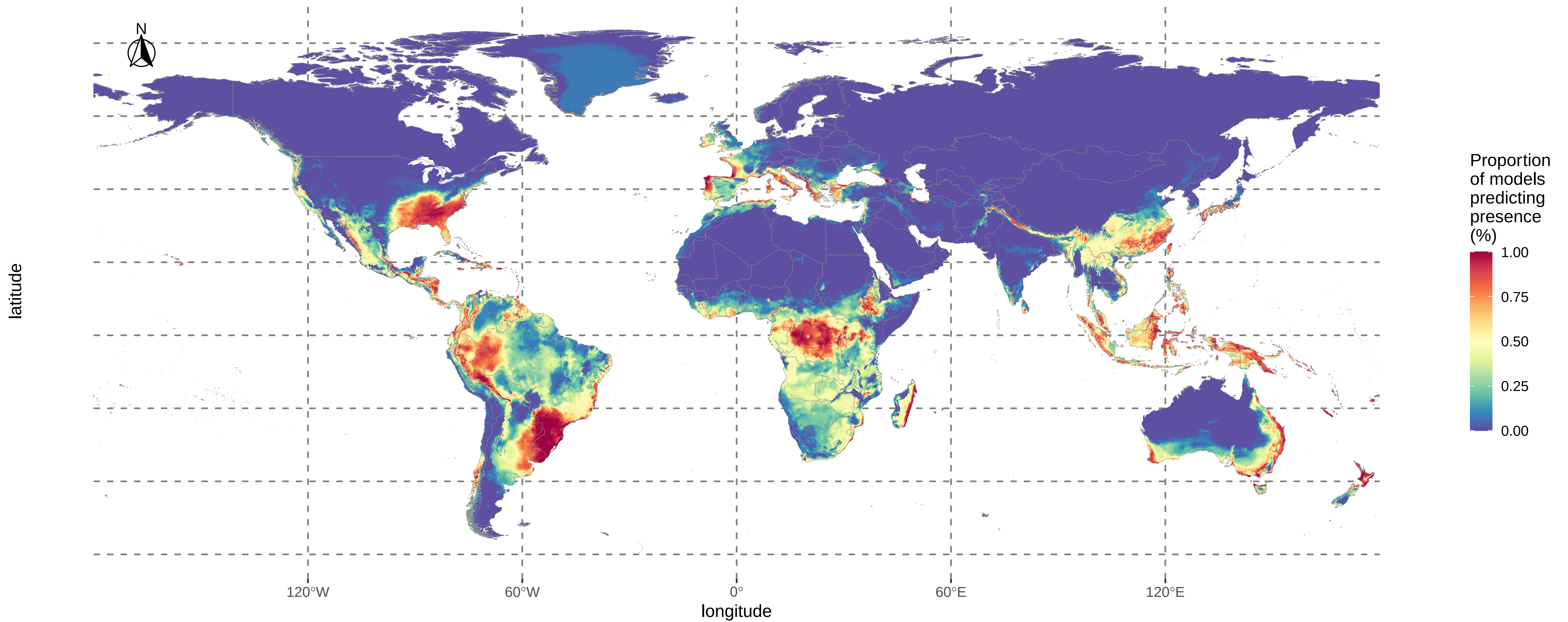

Figure S2−C: Consensus map showing the habitat suitability worldwide for Xylosandrus compactus in 2050 according to the RCP6.0.
Hot colours represent areas where the models reached a good agreement.

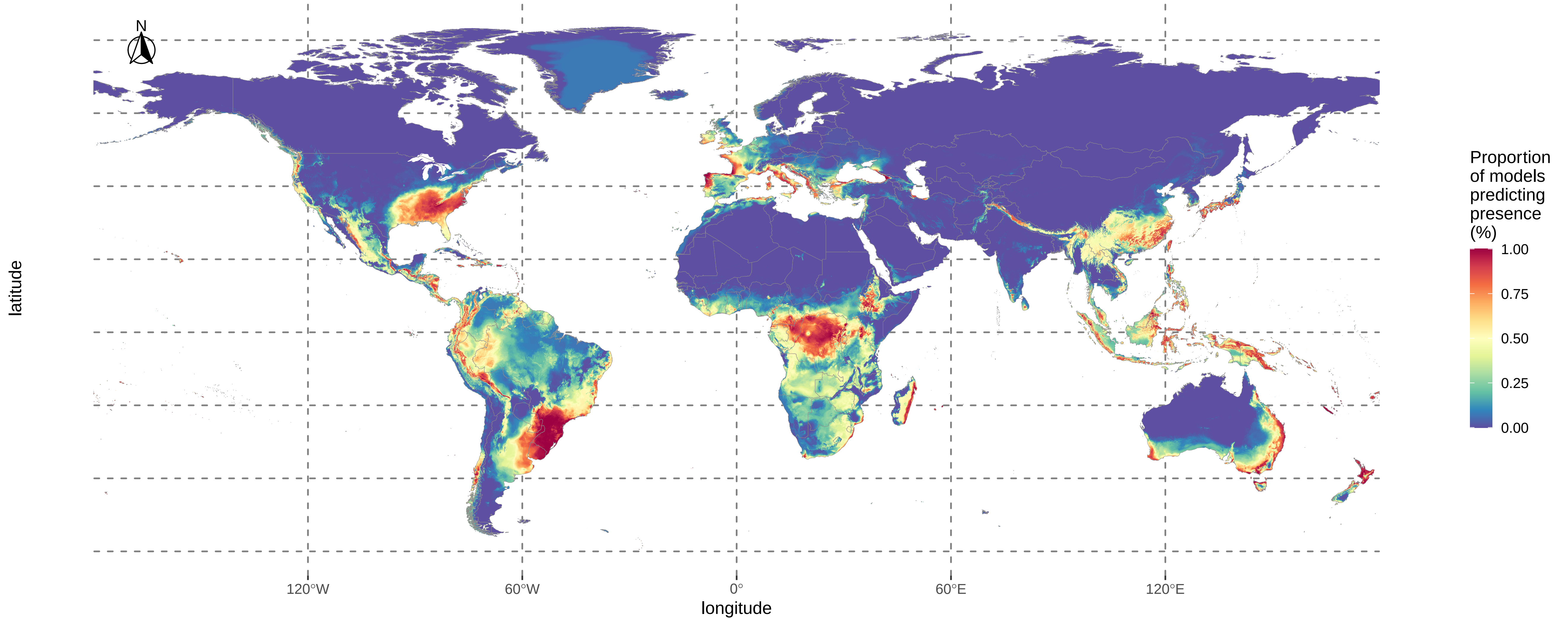

Figure S2−D: Consensus map showing the habitat suitability worldwide for Xylosandrus compactus in 2050 according to the RCP8.5.
Hot colours represent areas where the models reached a good agreement.

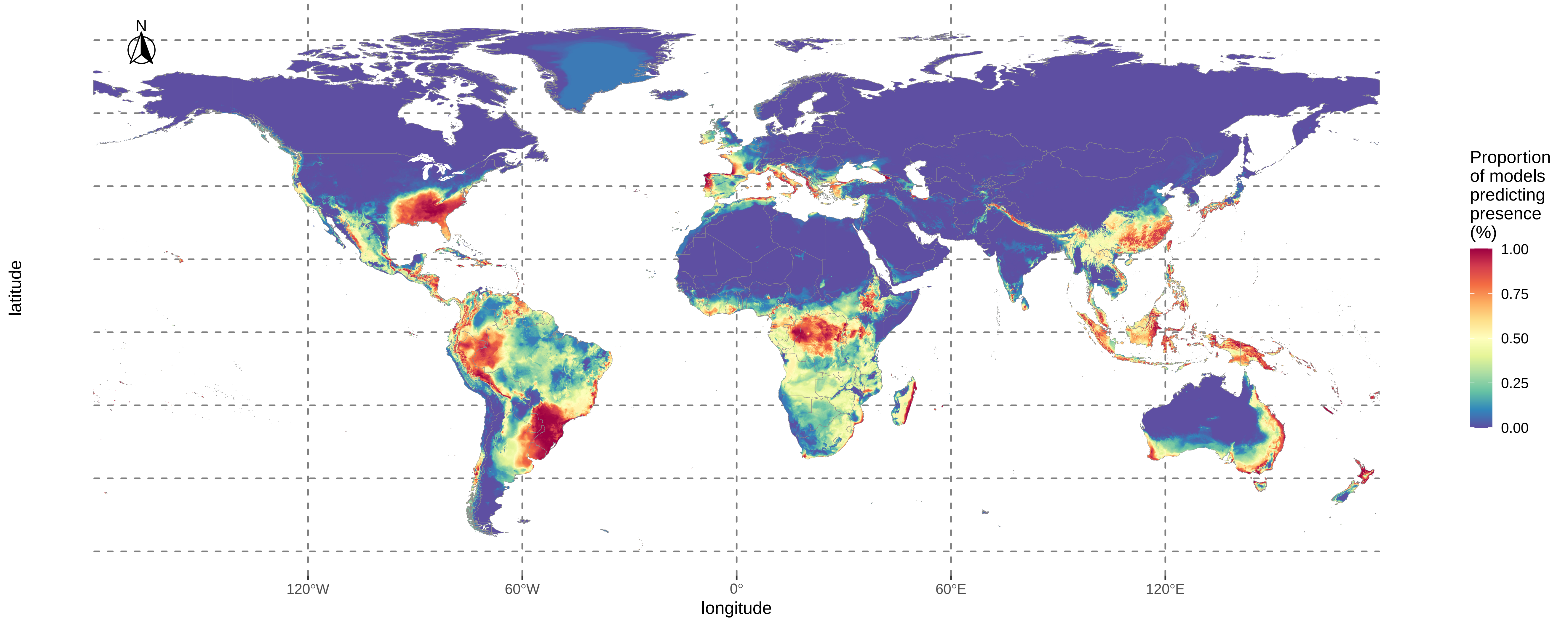

Figure S2−E: Consensus map showing the habitat suitability worldwide for Xylosandrus compactus in 2070 according to the RCP2.6.
Hot colours represent areas where the models reached a good agreement.

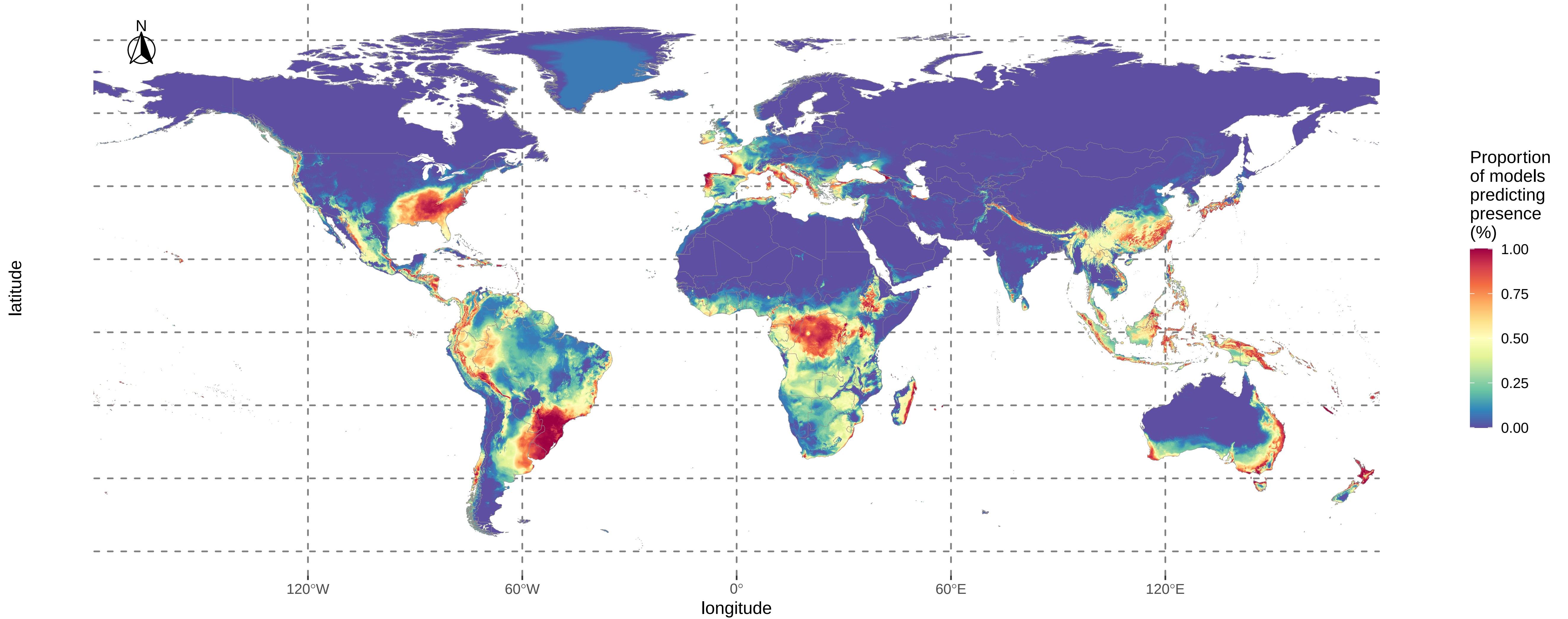

Figure S2−F: Consensus map showing the habitat suitability worldwide for Xylosandrus compactus in 2070 according to the RCP4.5.
Hot colours represent areas where the models reached a good agreement.

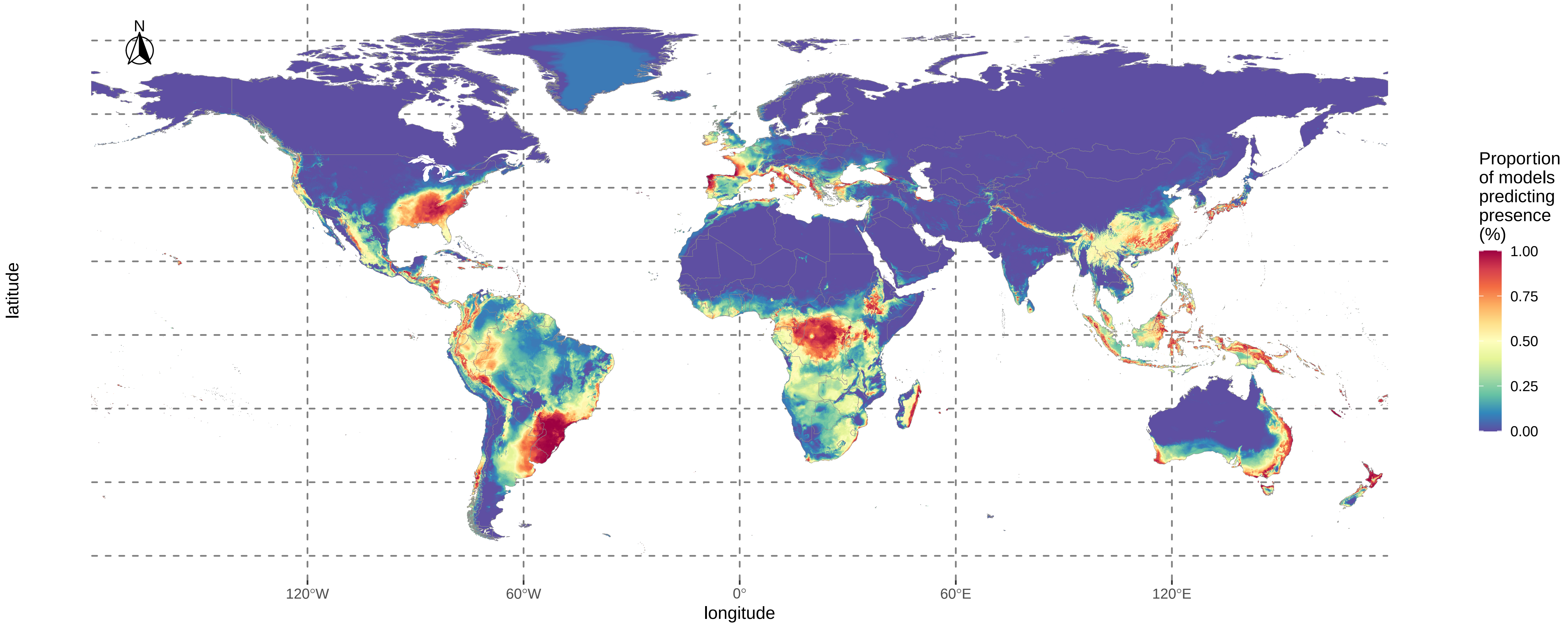

Figure S2−G: Consensus map showing the habitat suitability worldwide for Xylosandrus compactus in 2070 according to the RCP6.0.
Hot colours represent areas where the models reached a good agreement.

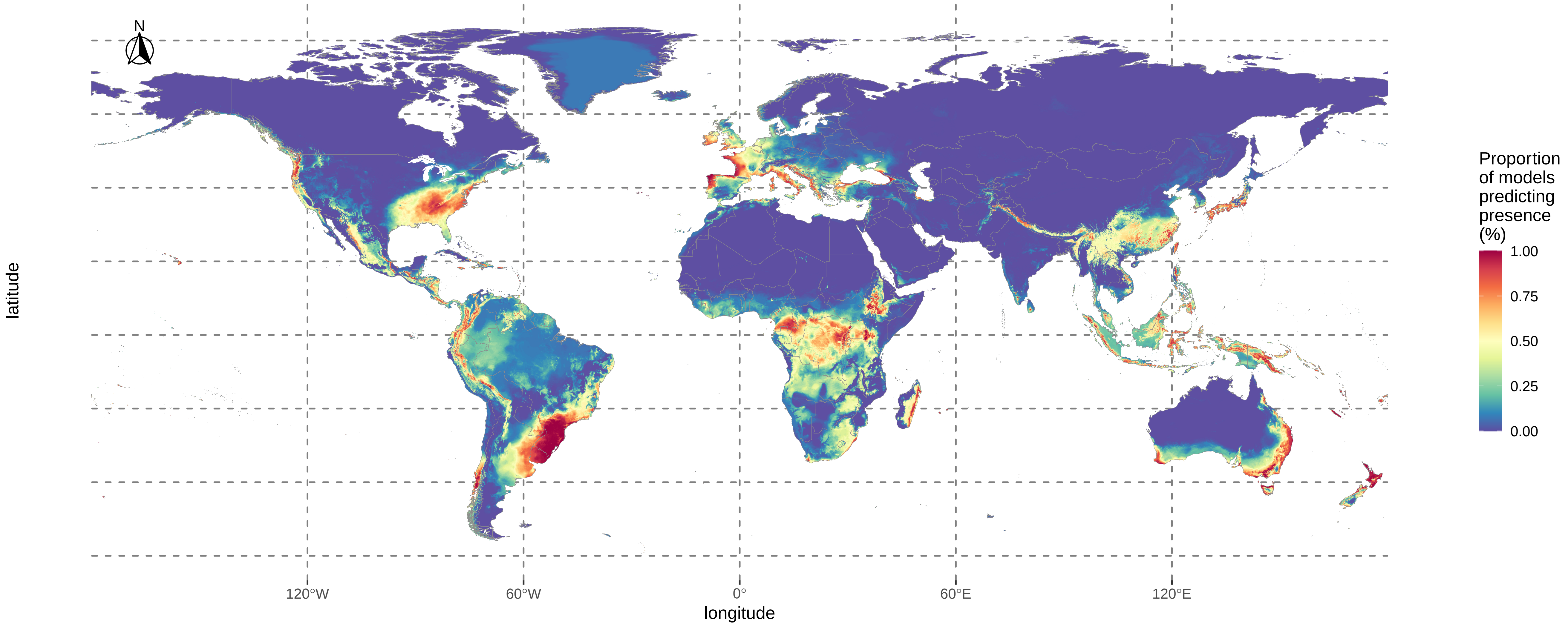

Figure S2−H: Consensus map showing the habitat suitability worldwide for Xylosandrus compactus in 2070 according to the RCP8.5.
Hot colours represent areas where the models reached a good agreement.
