# Supplementary material for: Climate change impact on the potential geographical distribution of two invading Xylosandrus ambrosia beetles
Source: Sci Rep. 2021 Jan 14;11:1339. doi: 10.1038/s41598-020-80157-9 (PMC7809213; doi:10.1038/s41598-020-80157-9)
Supplement: Supplementary file 3 — Supplementary Information 3. [file 41598_2020_80157_MOESM3_ESM.pdf]

Climate change impact on the potential geographical distribution of two invading *Xylosandrus* ambrosia beetles

T. Urvois, M.A. Auger-Rozenberg, A. Roques, J.P. Rossi, C. Kerdelhue

Figure S3: Maps showing the standard deviation of the habitat suitability worldwide for *Xylosandrus compactus* in 2050 and 2070 according to the RCPs 2.6, 4.5, 6.0 and 8.5. The maps were computed using R 4.0.0 (https://cran.r-project.org/) by calculating the standard deviation for each pixel of the presence−absence maps used to make the consensus maps. High values represent a low agreement between the models' predictions, whereas low values represent a high agreement between models' predictions.

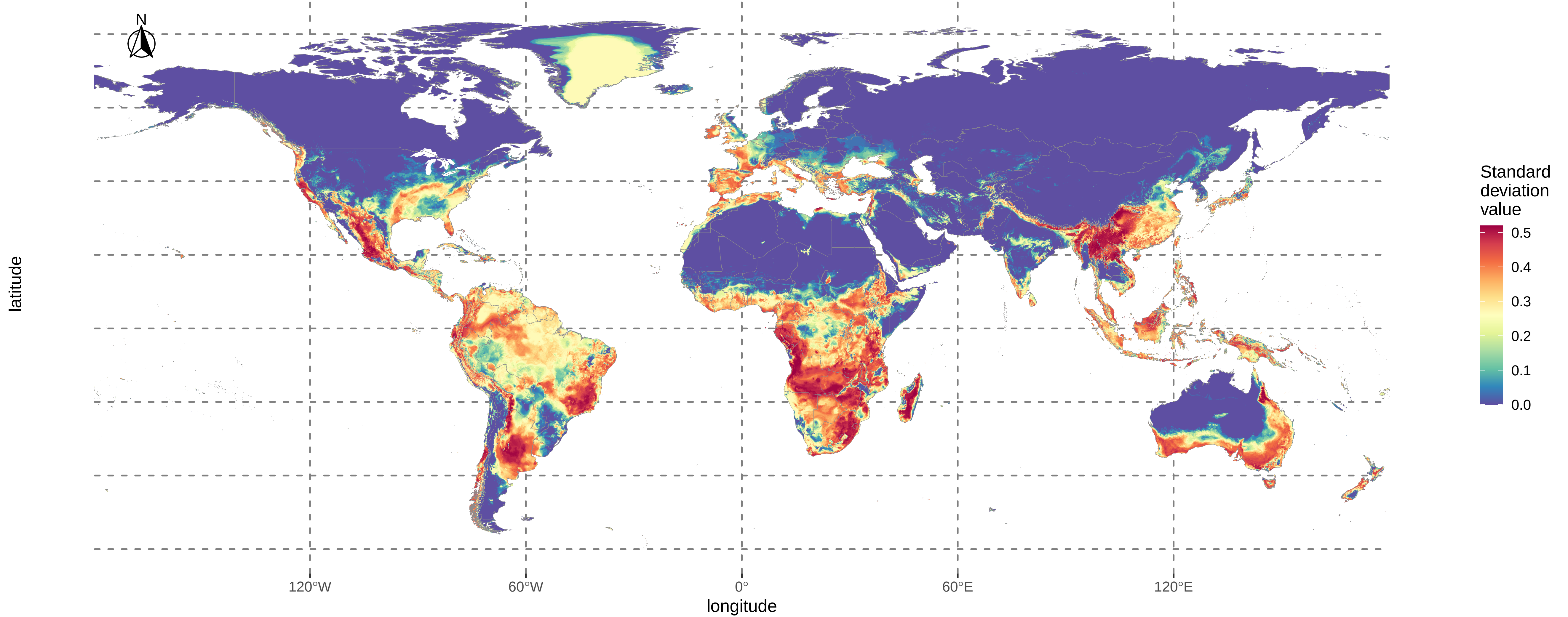

Figure S3−A: Map showing the standard deviation of the habitat suitability worldwide for Xylosandrus compactus in 2050 according to the RCP2.6.
This map was computed by calculating the standard deviation for each pixel of the presence−absence maps used to make the consensus map.
Hot colours represent areas with a high standard deviation.

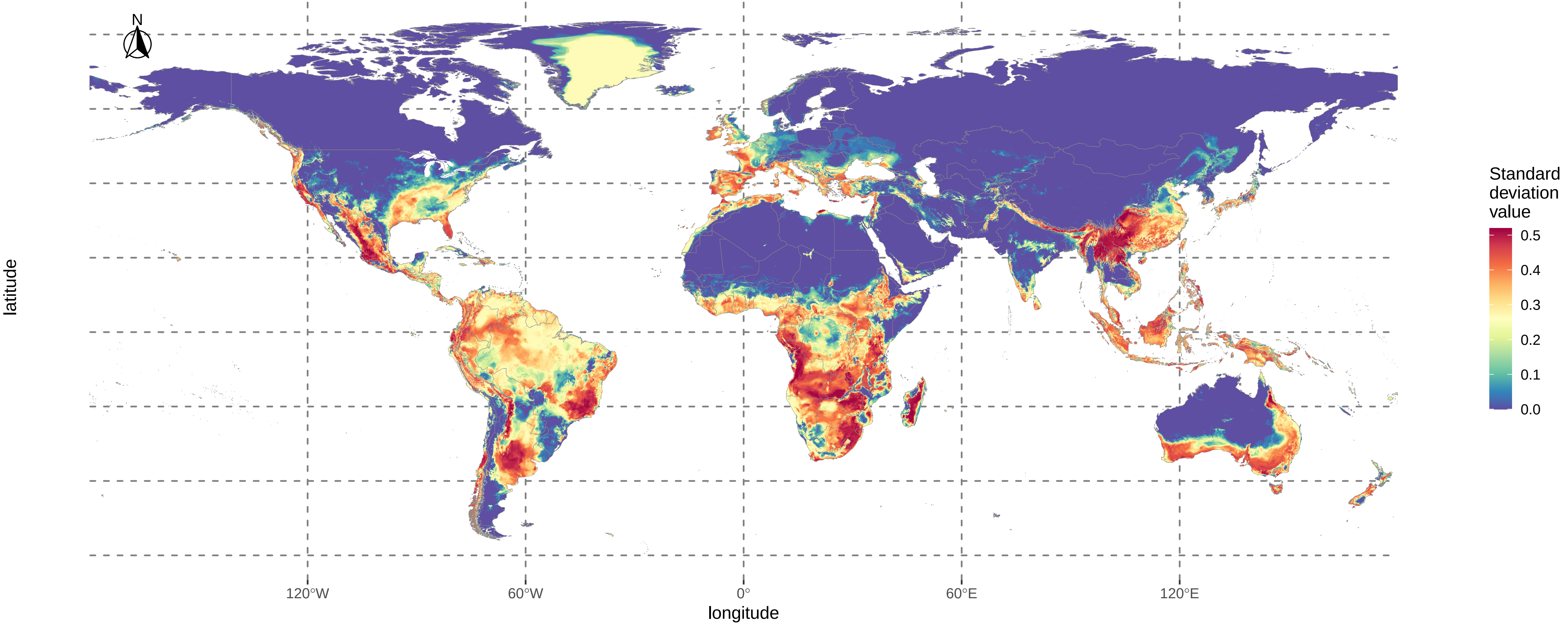

Figure S3−B: Map showing the standard deviation of the habitat suitability worldwide for Xylosandrus compactus in 2050 according to the RCP4.5.
This map was computed by calculating the standard deviation for each pixel of the presence−absence maps used to make the consensus map.
Hot colours represent areas with a high standard deviation.

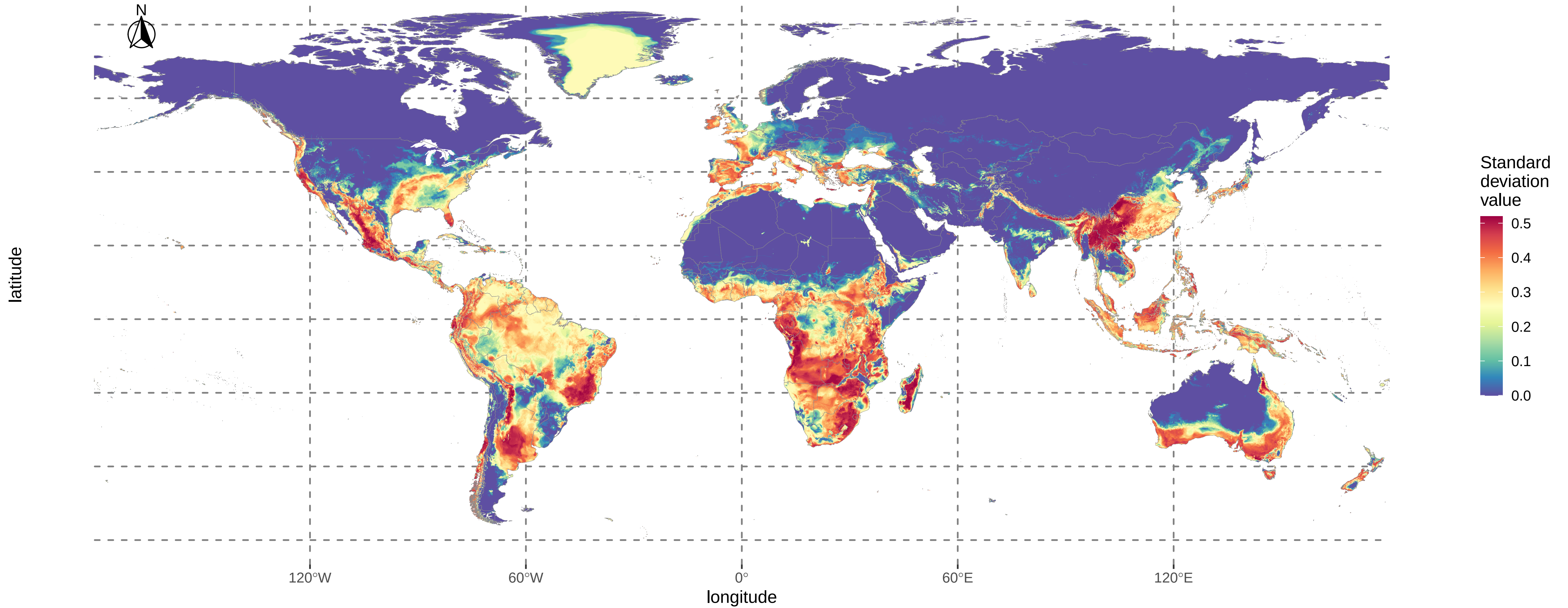

Figure S3−C: Map showing the standard deviation of the habitat suitability worldwide for Xylosandrus compactus in 2050 according to the RCP6.0.
This map was computed by calculating the standard deviation for each pixel of the presence−absence maps used to make the consensus map.
Hot colours represent areas with a high standard deviation.

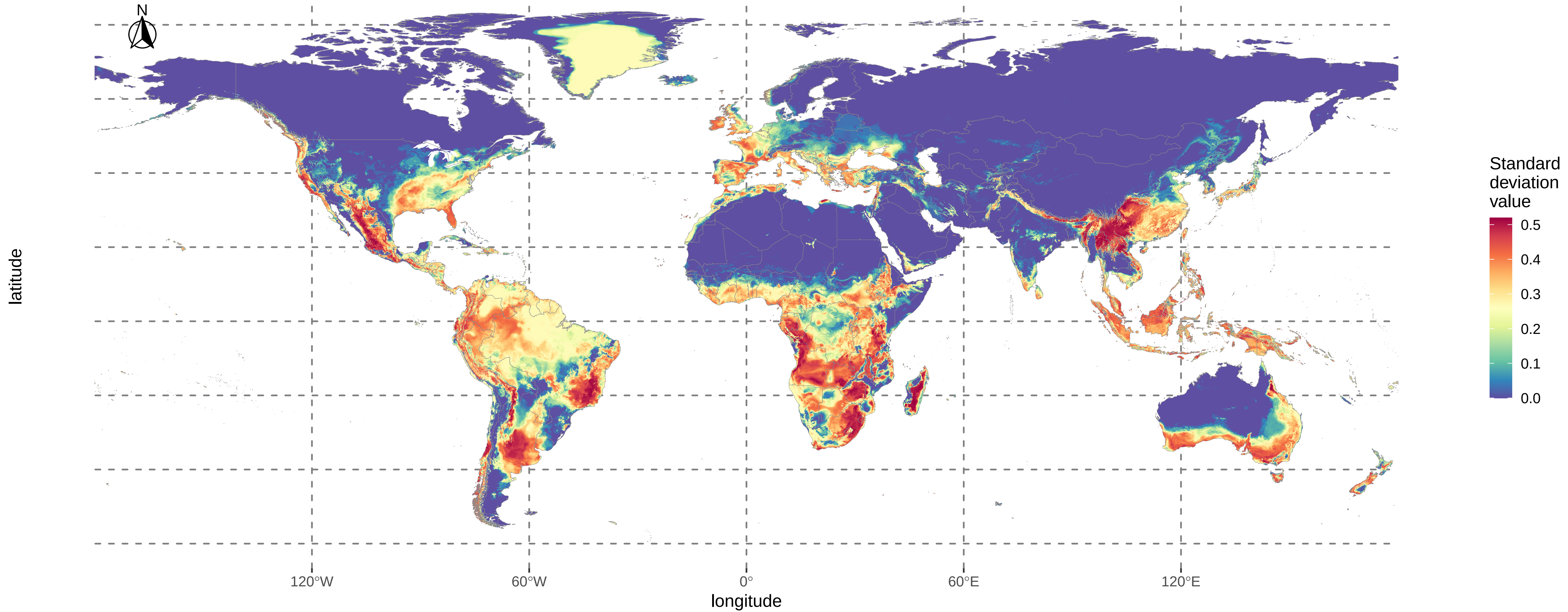

Figure S3−D: Map showing the standard deviation of the habitat suitability worldwide for Xylosandrus compactus in 2050 according to the RCP8.5.
This map was computed by calculating the standard deviation for each pixel of the presence−absence maps used to make the consensus map.
Hot colours represent areas with a high standard deviation.

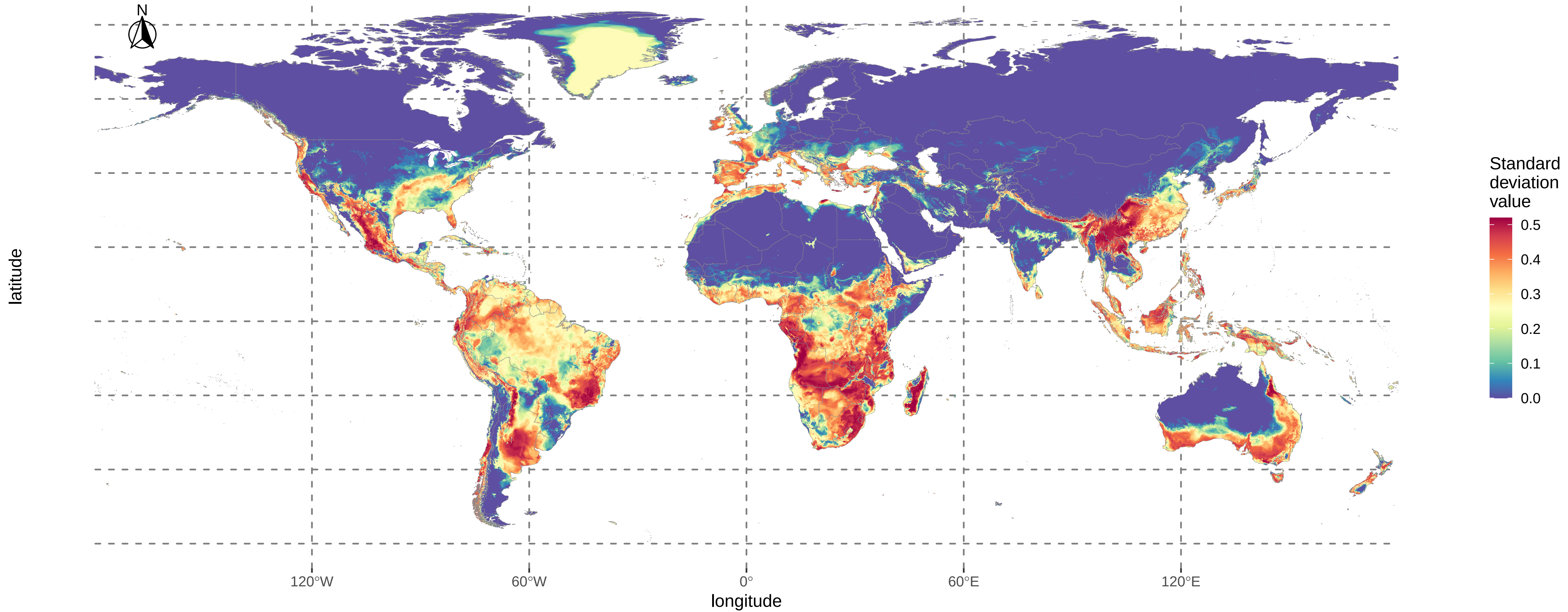

Figure S3−E: Map showing the standard deviation of the habitat suitability worldwide for Xylosandrus compactus in 2070 according to the RCP2.6.
This map was computed by calculating the standard deviation for each pixel of the presence−absence maps used to make the consensus map.
Hot colours represent areas with a high standard deviation.

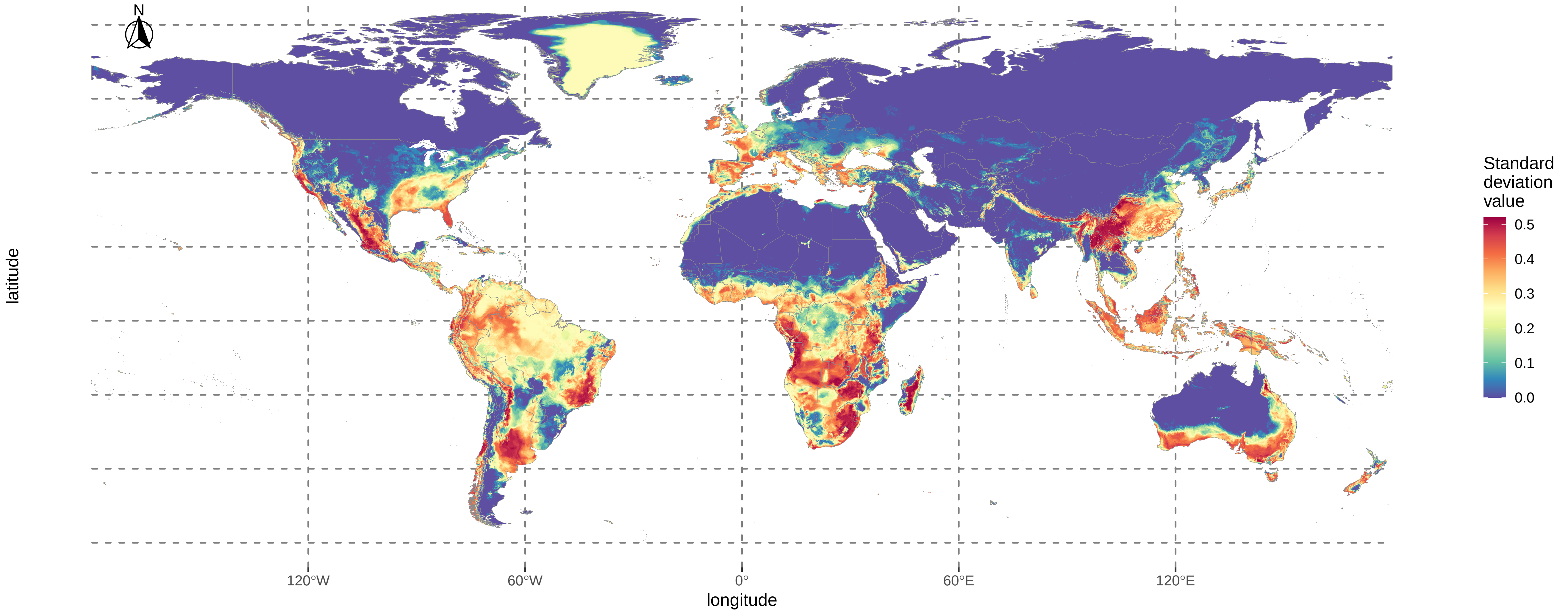

Figure S3−F: Map showing the standard deviation of the habitat suitability worldwide for Xylosandrus compactus in 2070 according to the RCP4.5.
This map was computed by calculating the standard deviation for each pixel of the presence−absence maps used to make the consensus map.
Hot colours represent areas with a high standard deviation.

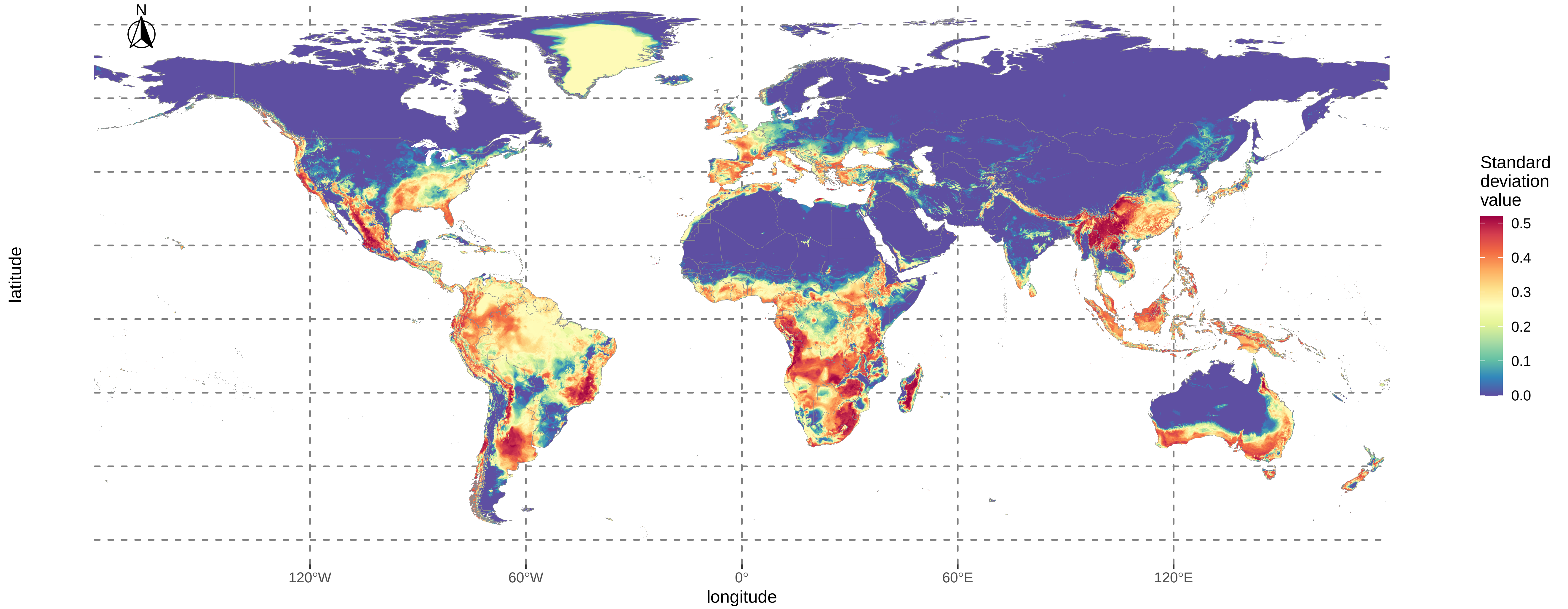

Figure S3−G: Map showing the standard deviation of the habitat suitability worldwide for Xylosandrus compactus in 2070 according to the RCP6.0.
This map was computed by calculating the standard deviation for each pixel of the presence−absence maps used to make the consensus map.
Hot colours represent areas with a high standard deviation.

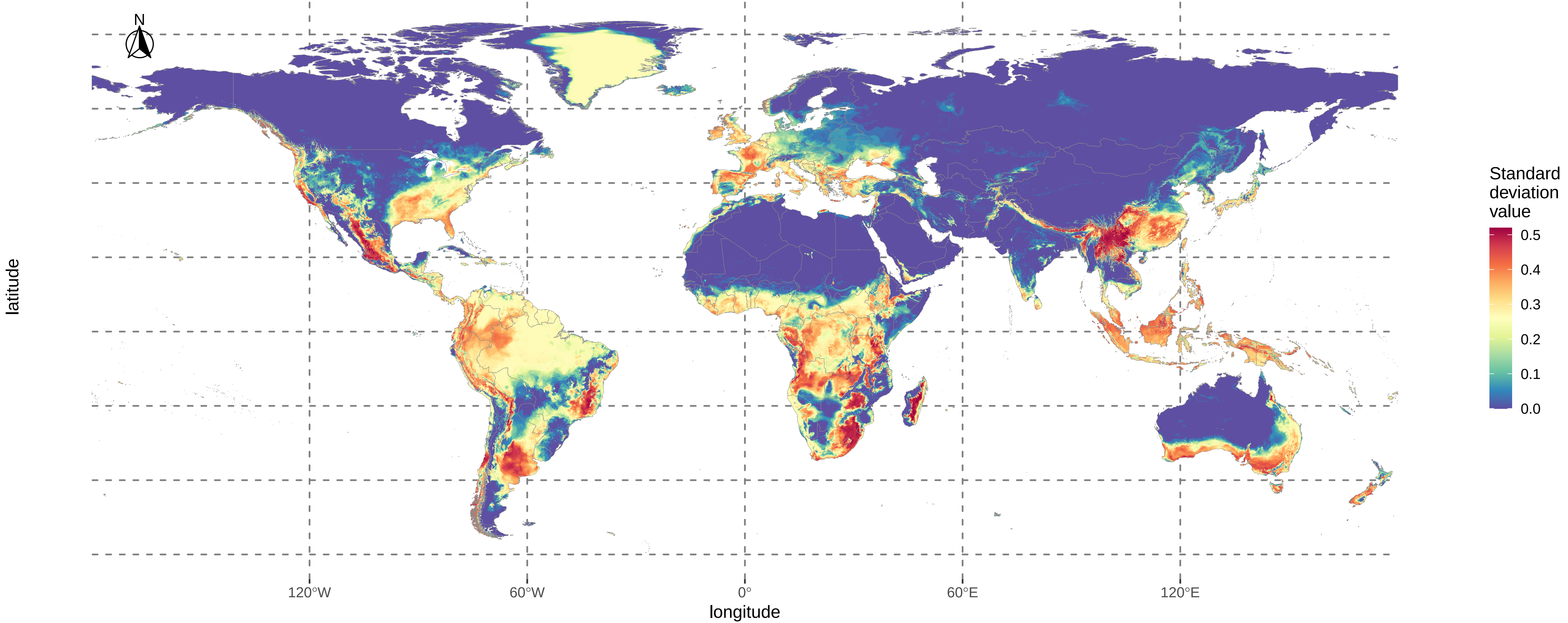

Figure S3−H: Map showing the standard deviation of the habitat suitability worldwide for Xylosandrus compactus in 2070 according to the RCP8.5.
This map was computed by calculating the standard deviation for each pixel of the presence−absence maps used to make the consensus map.
Hot colours represent areas with a high standard deviation.
