# Supplementary material for: Climate change impact on the potential geographical distribution of two invading Xylosandrus ambrosia beetles
Source: Sci Rep. 2021 Jan 14;11:1339. doi: 10.1038/s41598-020-80157-9 (PMC7809213; doi:10.1038/s41598-020-80157-9)
Supplement: Supplementary file 9 — Supplementary Information 9. [file 41598_2020_80157_MOESM9_ESM.pdf]

Climate change impact on the potential geographical distribution of two invading *Xylosandrus* ambrosia beetles

T. Urvois, M.A. Auger-Rozenberg, A. Roques, J.P. Rossi, C. Kerdelhue

Figure S4.6: Maps illustrating the habitat suitability worldwide for *Xylosandrus compactus* in 2070 for the RCP4.5 for the different GCM (BC, CC, GS, HD, HE, IP, MI, MR, MC, MG, NO). The values represent the percentage of models predicting each pixel as suitable. The maps were generated using R 4.0.0 (https://cran.r-project.org/).

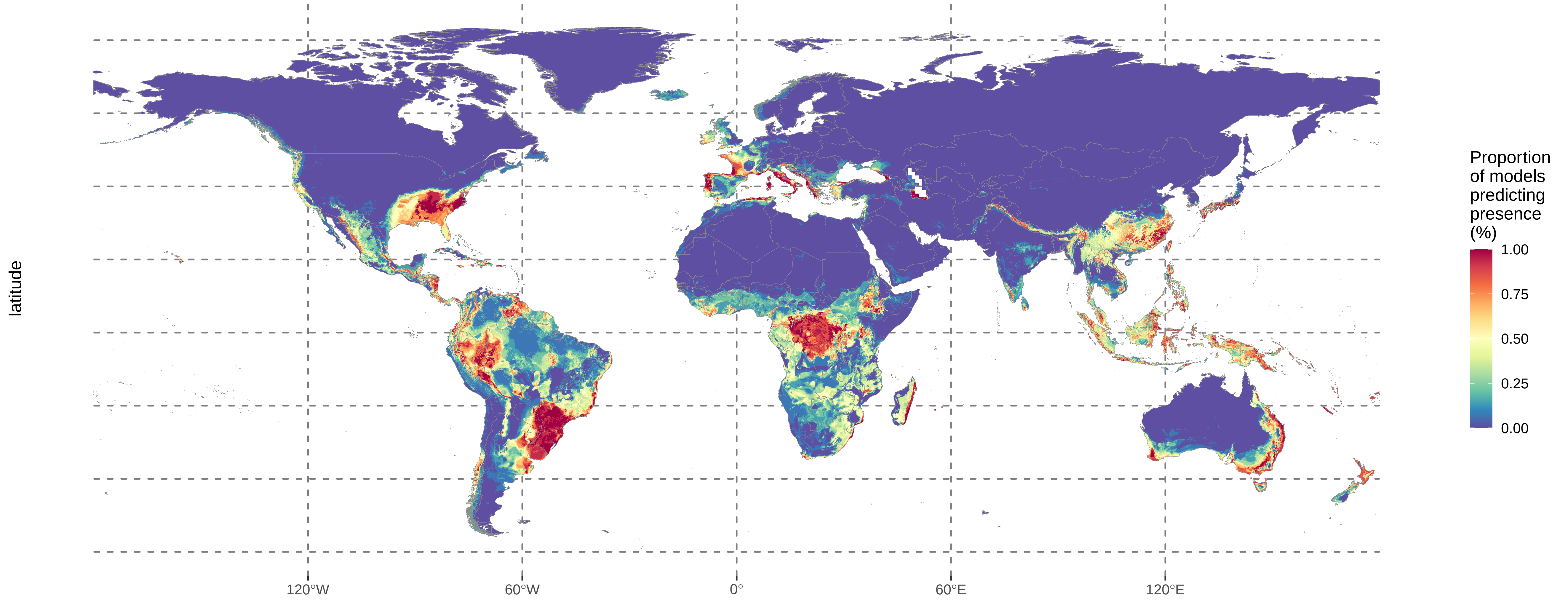

Map illustrating the habitat suitability for the GCM BC in 2070 for the RCP 4.5.
Hot colours represent a high suitability, whereas cold colours represent low suitability.

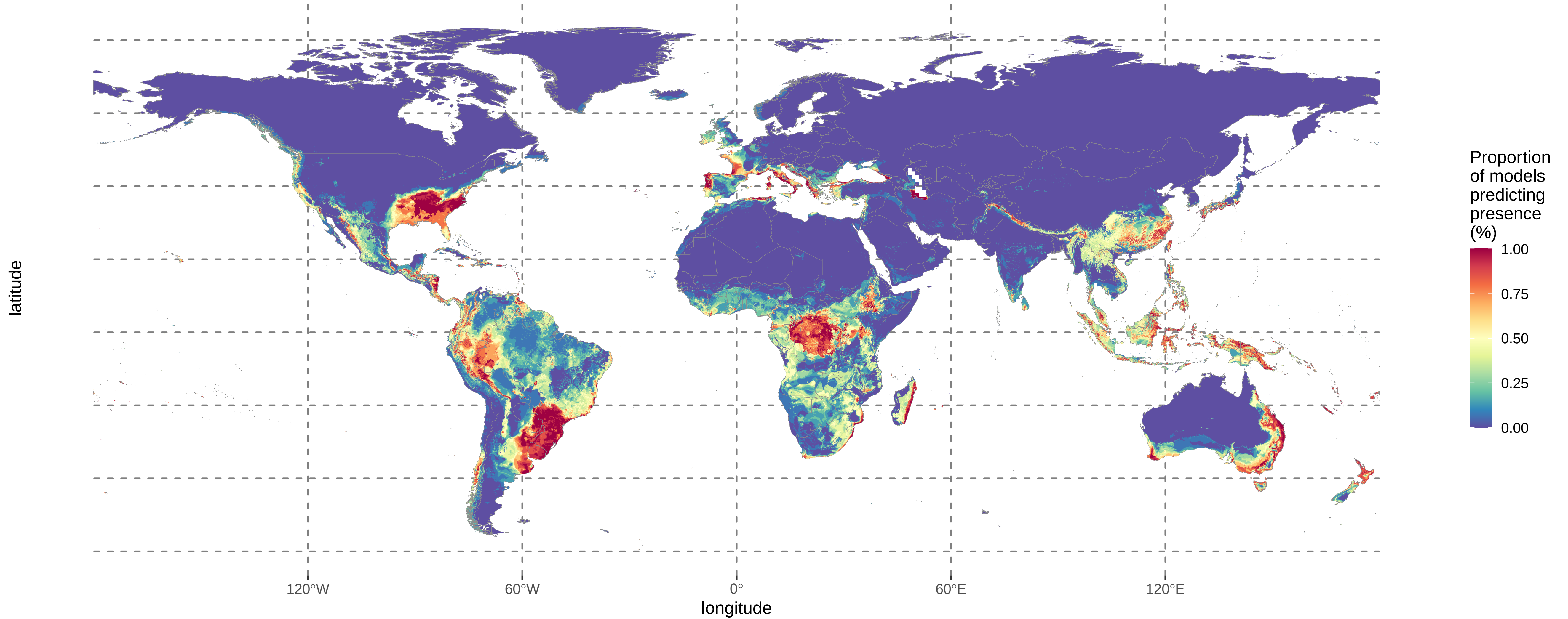

Map illustrating the habitat suitability for the GCM CC in 2070 for the RCP 4.5.
Hot colours represent a high suitability, whereas cold colours represent low suitability.

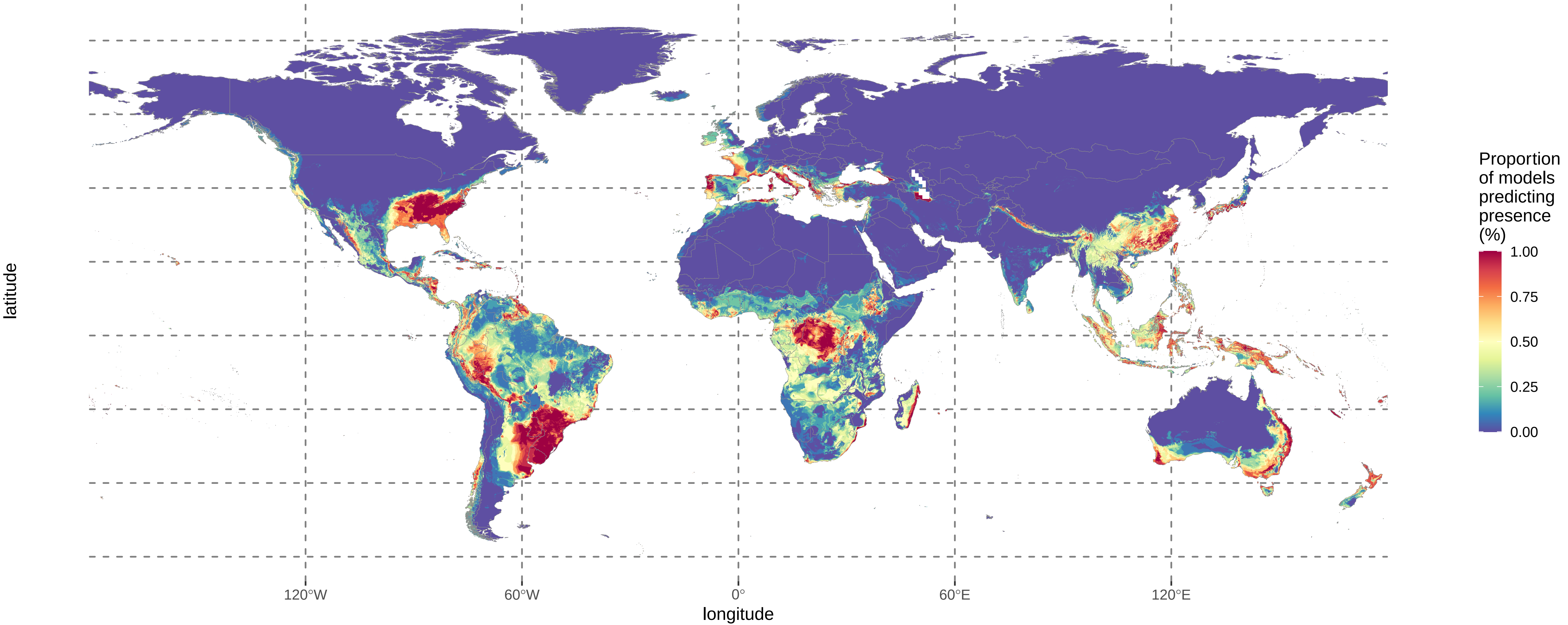

Map illustrating the habitat suitability for the GCM GS in 2070 for the RCP 4.5.
Hot colours represent a high suitability, whereas cold colours represent low suitability.

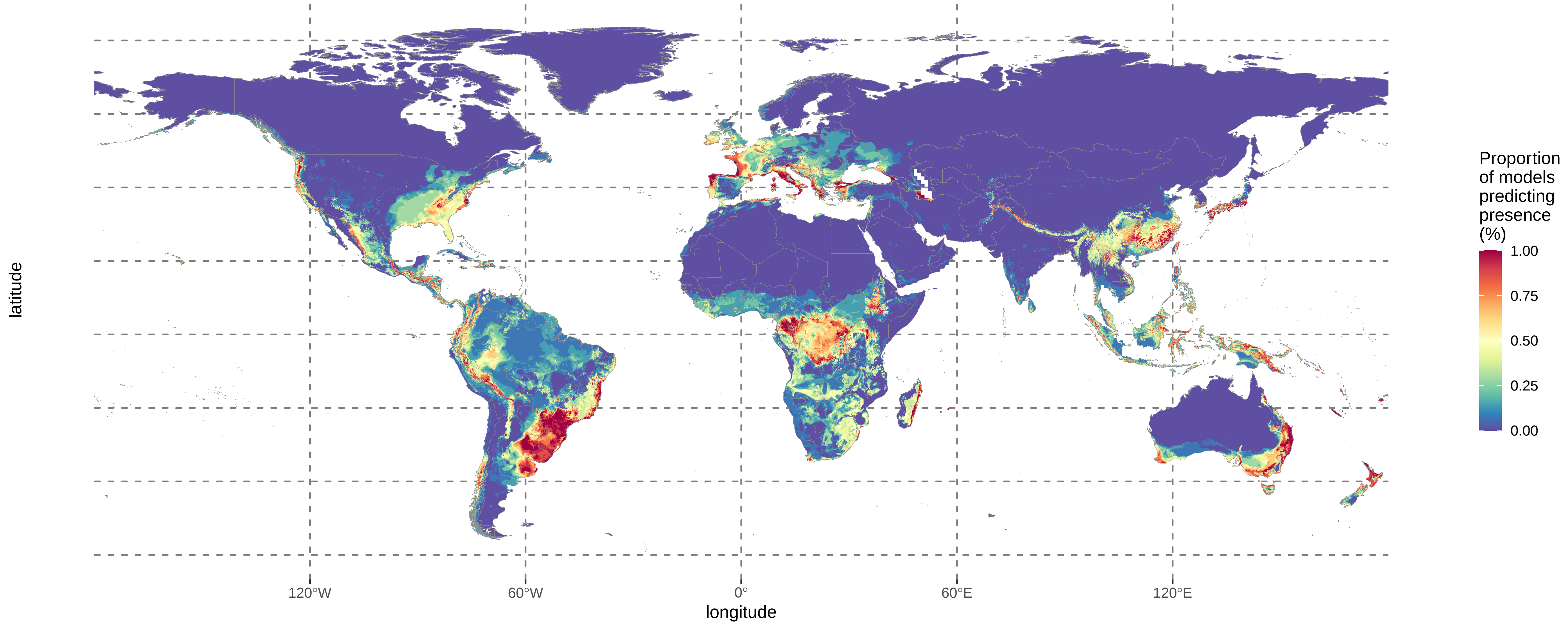

Map illustrating the habitat suitability for the GCM HD in 2070 for the RCP 4.5.
Hot colours represent a high suitability, whereas cold colours represent low suitability.

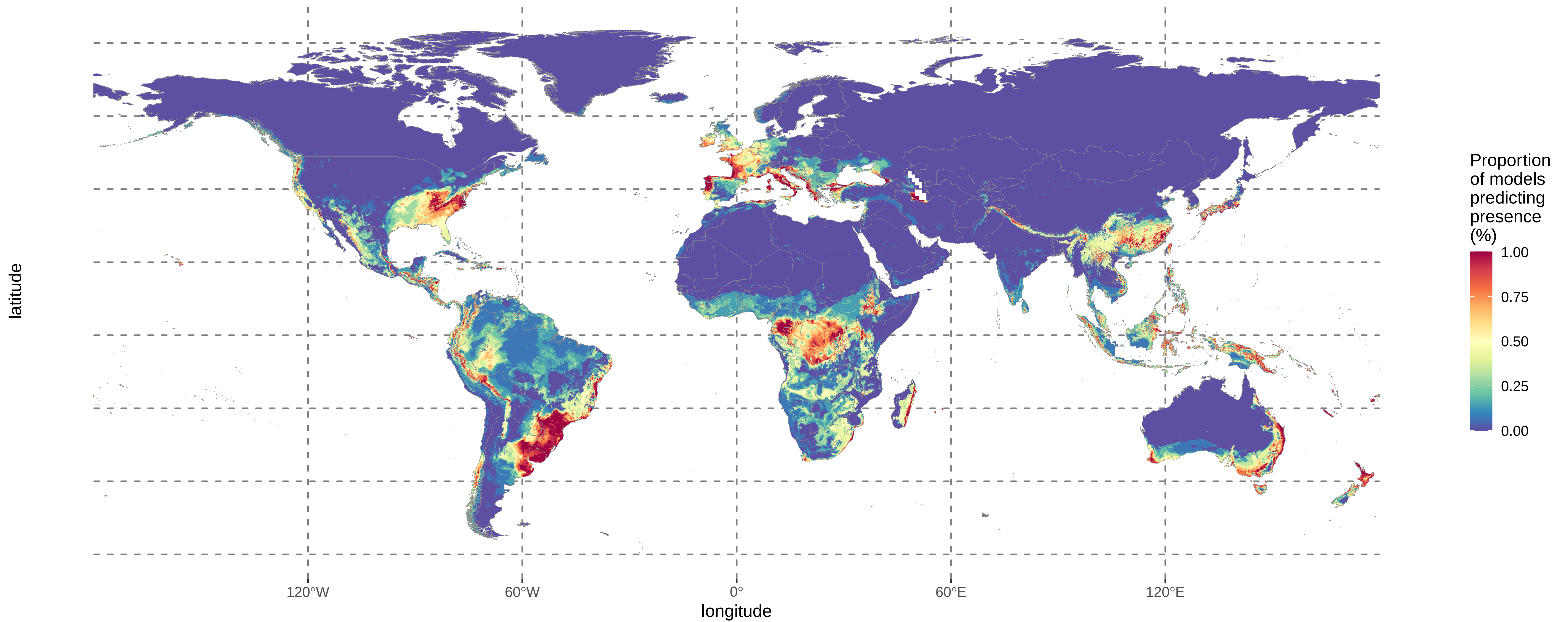

Map illustrating the habitat suitability for the GCM HE in 2070 for the RCP 4.5.
Hot colours represent a high suitability, whereas cold colours represent low suitability.

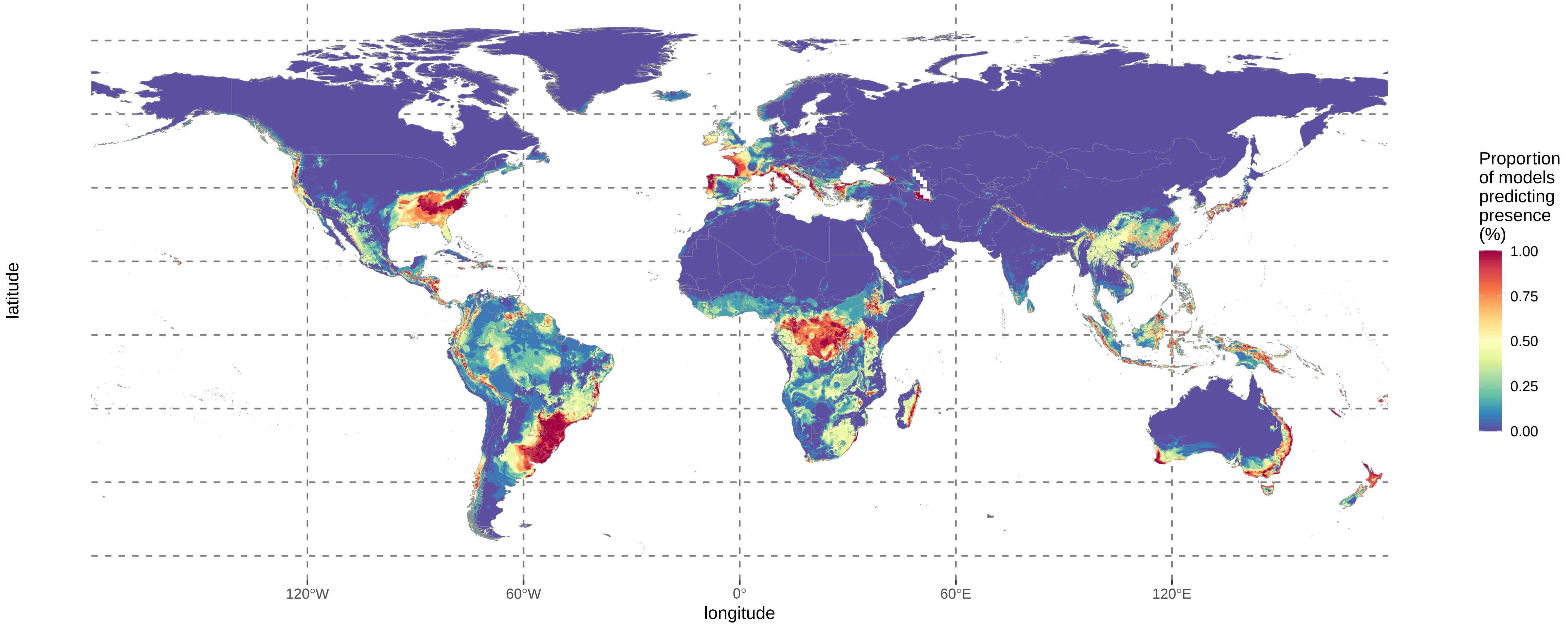

Map illustrating the habitat suitability for the GCM IP in 2070 for the RCP 4.5.
Hot colours represent a high suitability, whereas cold colours represent low suitability.

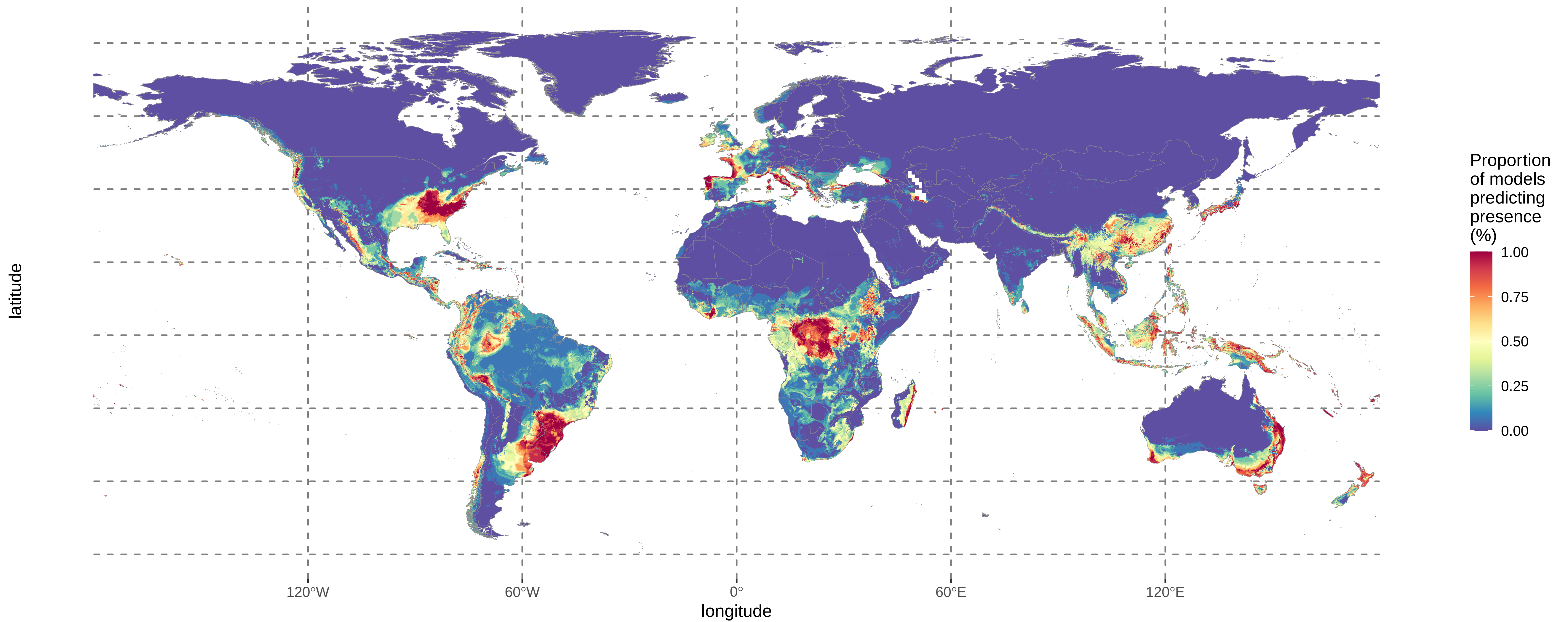

Map illustrating the habitat suitability for the GCM MI in 2070 for the RCP 4.5.
Hot colours represent a high suitability, whereas cold colours represent low suitability.

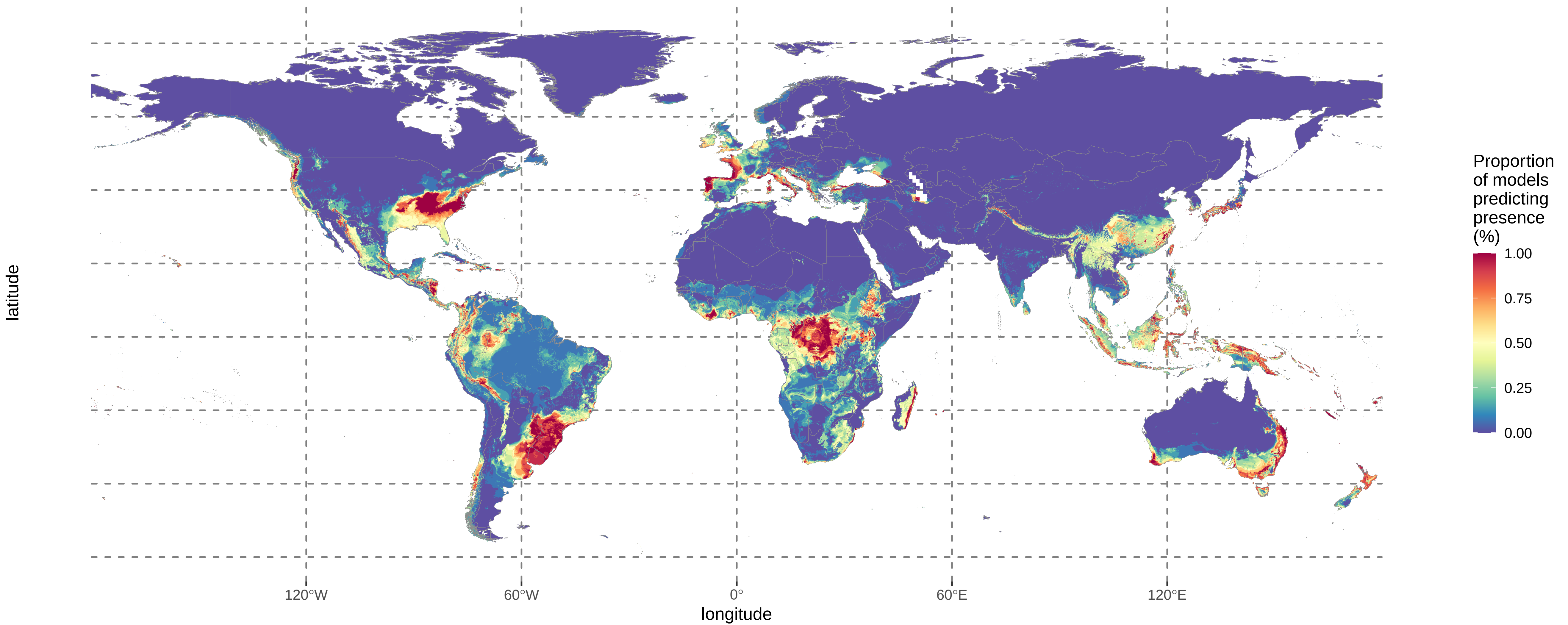

Map illustrating the habitat suitability for the GCM MR in 2070 for the RCP 4.5.
Hot colours represent a high suitability, whereas cold colours represent low suitability.

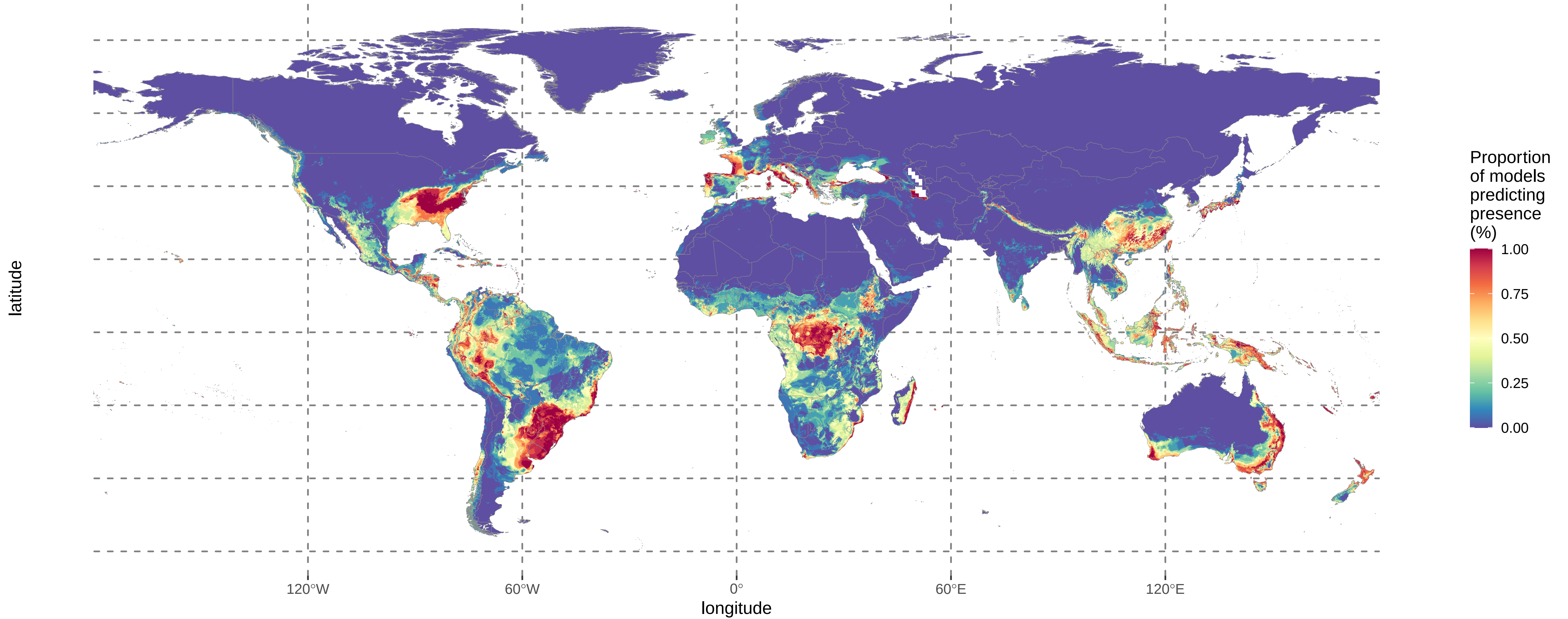

Map illustrating the habitat suitability for the GCM MC in 2070 for the RCP 4.5.
Hot colours represent a high suitability, whereas cold colours represent low suitability.

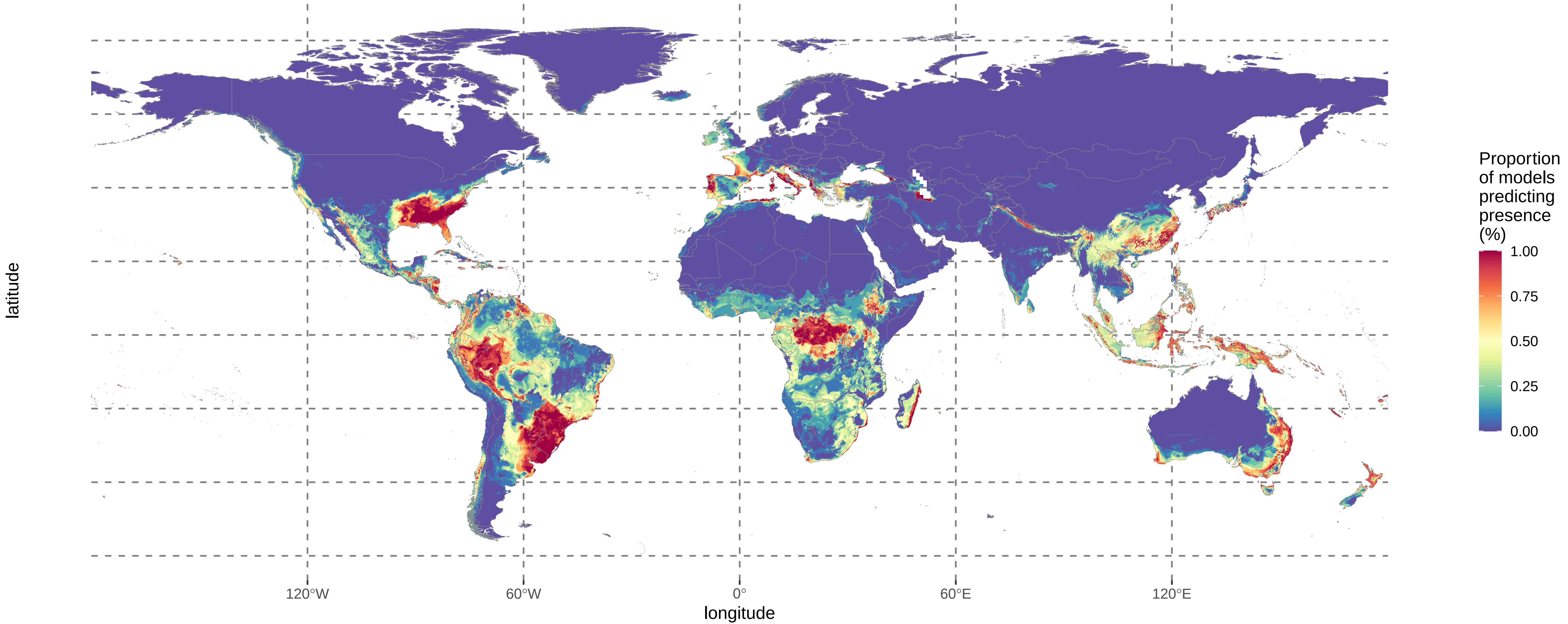

Map illustrating the habitat suitability for the GCM MG in 2070 for the RCP 4.5.
Hot colours represent a high suitability, whereas cold colours represent low suitability.

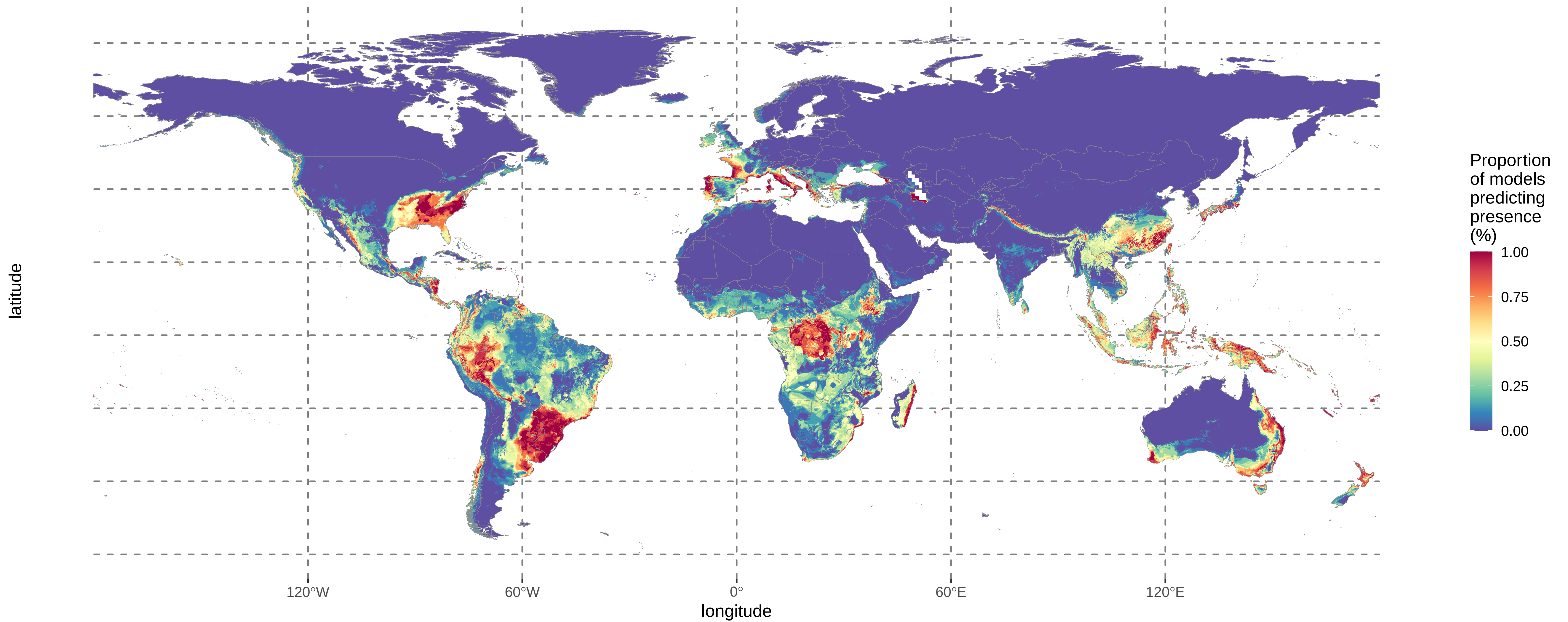

Map illustrating the habitat suitability for the GCM NO in 2070 for the RCP 4.5.
Hot colours represent a high suitability, whereas cold colours represent low suitability.
