# Supplementary material for: Climate change impact on the potential geographical distribution of two invading Xylosandrus ambrosia beetles
Source: Sci Rep. 2021 Jan 14;11:1339. doi: 10.1038/s41598-020-80157-9 (PMC7809213; doi:10.1038/s41598-020-80157-9)
Supplement: Supplementary file 19 — Supplementary Information 19. [file 41598_2020_80157_MOESM19_ESM.pdf]

Climate change impact on the potential geographical distribution of two invading *Xylosandrus* ambrosia beetles

T. Urvois, M.A. Auger-Rozenberg, A. Roques, J.P. Rossi, C. Kerdelhue

Figure S5.8: Maps illustrating the centered values for the different GCM (BC, CC, GS, HD, HE, IP, MI, MR, MC, MG, NO) in 2070 for the RCP8.5. The maps display a negative value when the GCM considered predicts lower suitability than the average of all GCMs and a positive value otherwise. The maps were generated using R 4.0.0 (https://cran.r-project.org/).

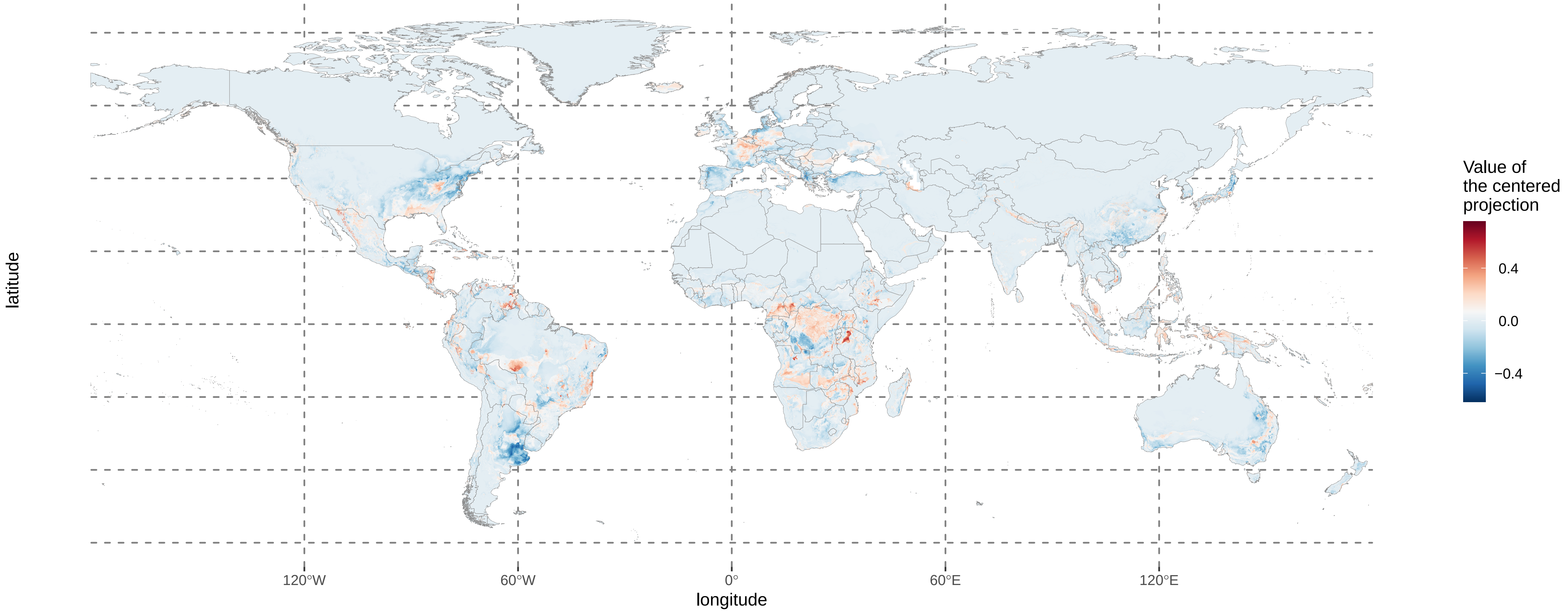

Map illustrating the centered values for the GCM BC in 2070 for the RCP 8.5.
This map was computed by substracting the average value for each pixel to the value for the GCM BC.
Hot colours represent a value higher than average, whereas cold colours represent values lower than average.

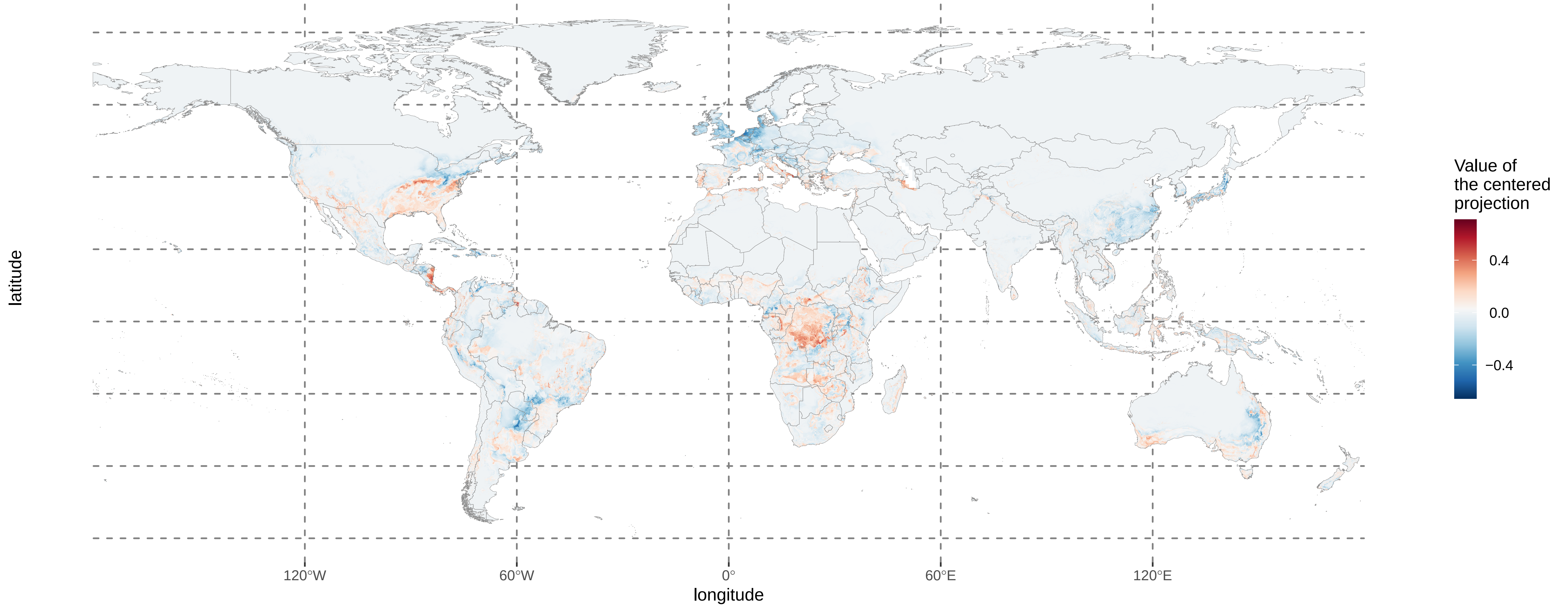

Map illustrating the centered values for the GCM CC in 2070 for the RCP 8.5.
This map was computed by substracting the average value for each pixel to the value for the GCM CC.
Hot colours represent a value higher than average, whereas cold colours represent values lower than average.

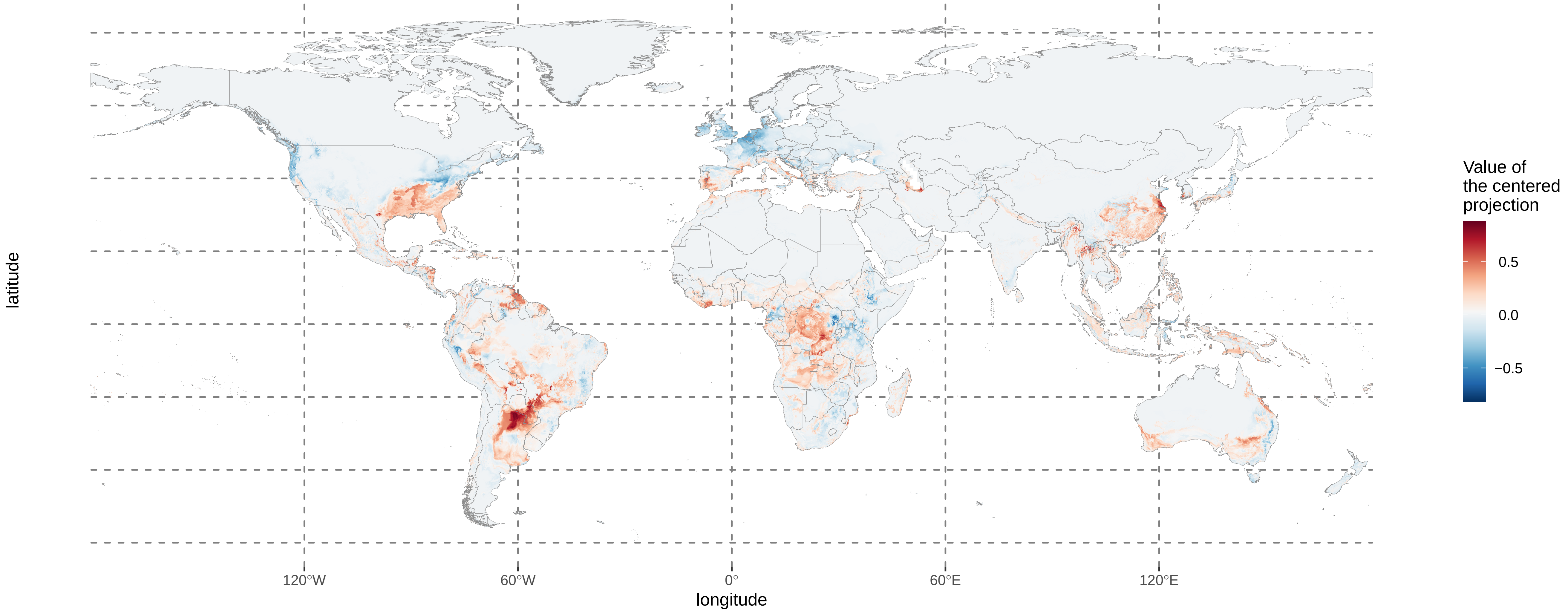

Map illustrating the centered values for the GCM GS in 2070 for the RCP 8.5.
This map was computed by substracting the average value for each pixel to the value for the GCM GS.
Hot colours represent a value higher than average, whereas cold colours represent values lower than average.

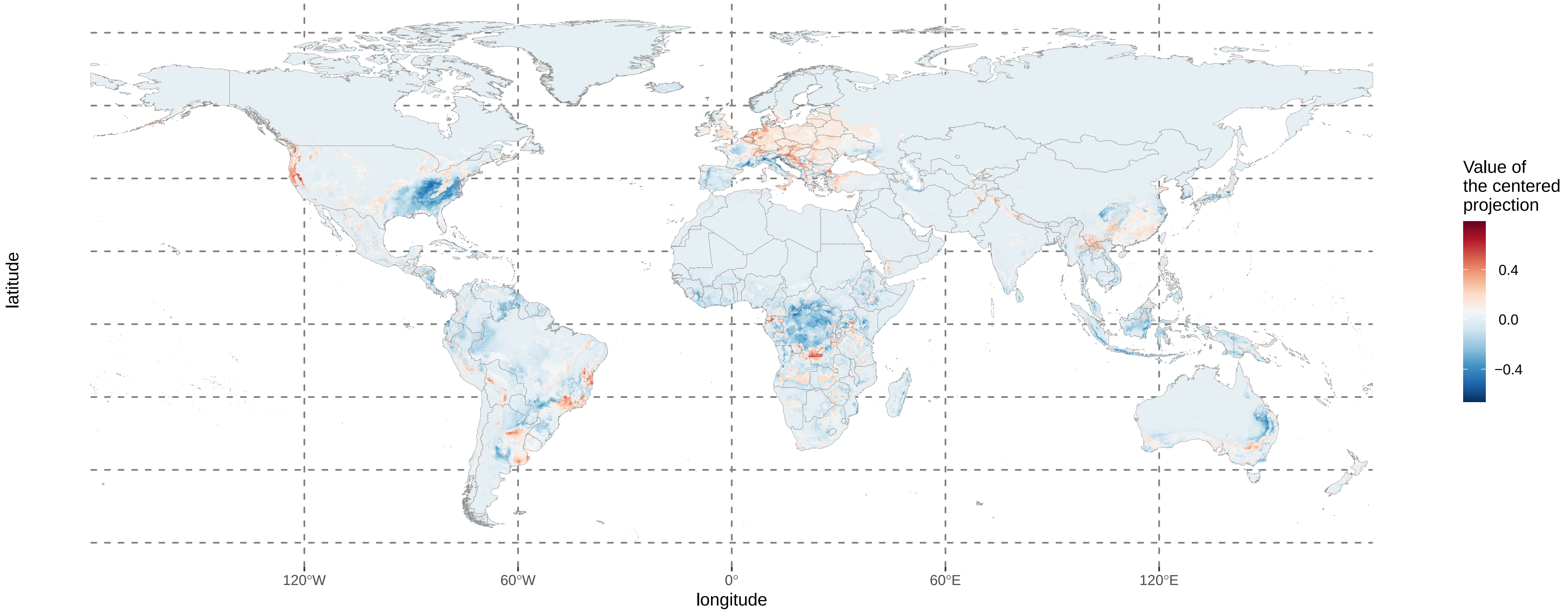

Map illustrating the centered values for the GCM HD in 2070 for the RCP 8.5.
This map was computed by substracting the average value for each pixel to the value for the GCM HD.
Hot colours represent a value higher than average, whereas cold colours represent values lower than average.

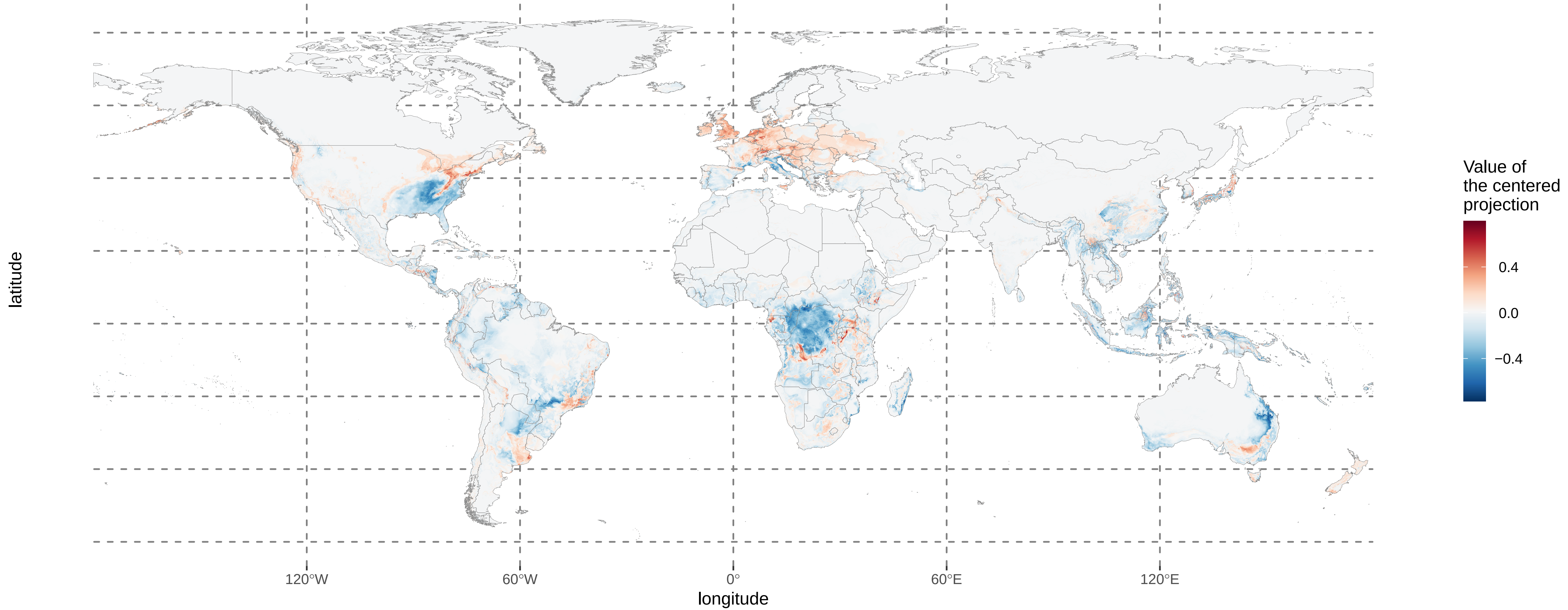

Map illustrating the centered values for the GCM HE in 2070 for the RCP 8.5.
This map was computed by substracting the average value for each pixel to the value for the GCM HE.
Hot colours represent a value higher than average, whereas cold colours represent values lower than average.

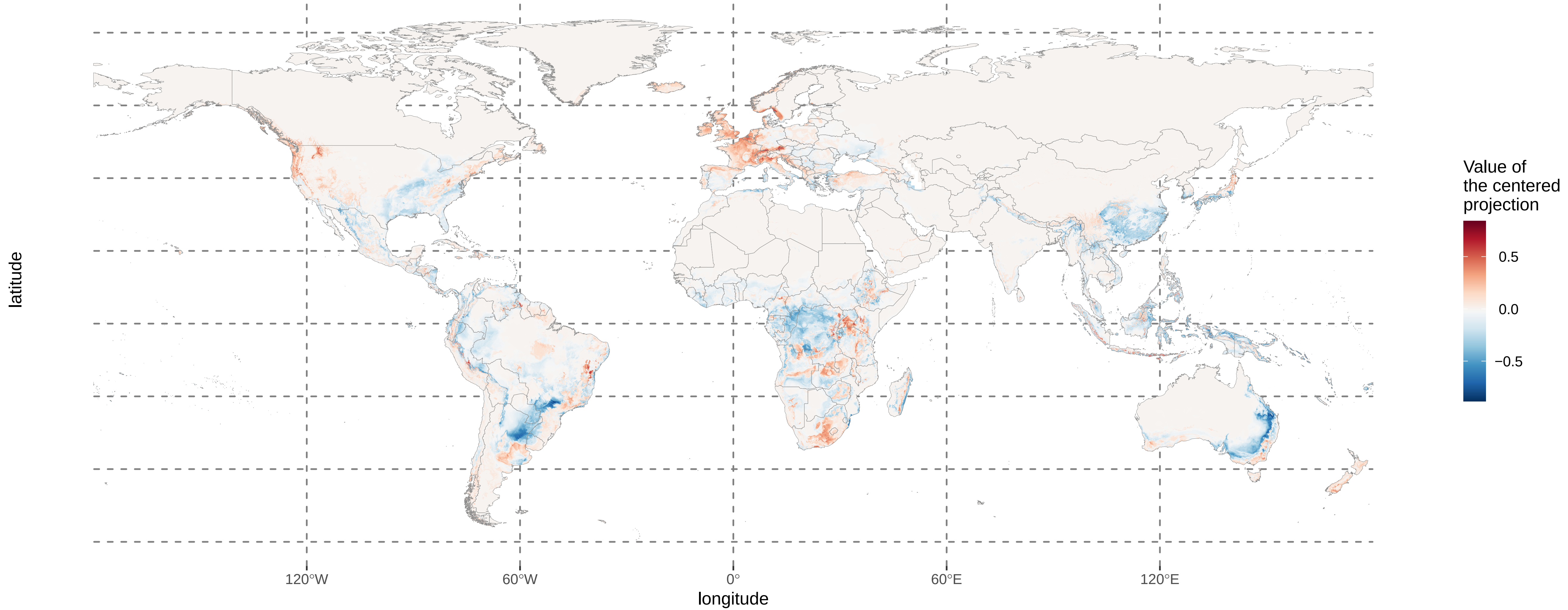

Map illustrating the centered values for the GCM IP in 2070 for the RCP 8.5.
This map was computed by substracting the average value for each pixel to the value for the GCM IP.
Hot colours represent a value higher than average, whereas cold colours represent values lower than average.

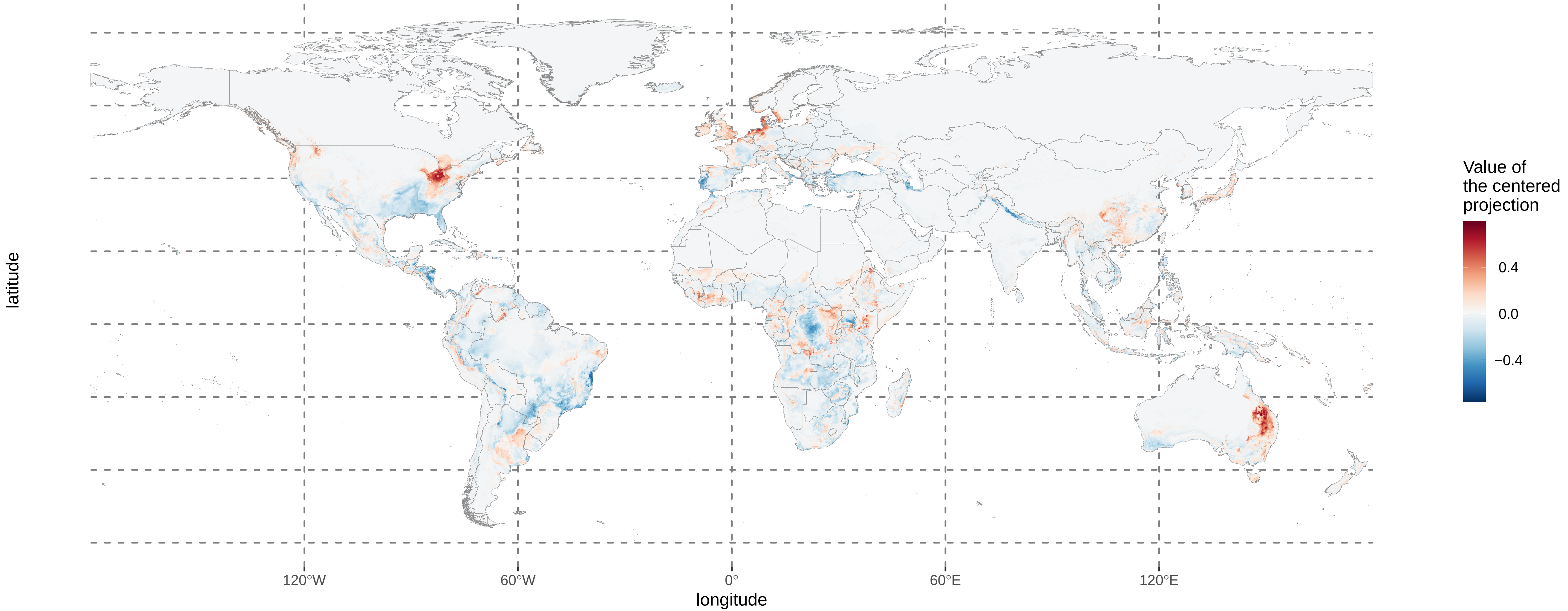

Map illustrating the centered values for the GCM MI in 2070 for the RCP 8.5.
This map was computed by substracting the average value for each pixel to the value for the GCM MI.
Hot colours represent a value higher than average, whereas cold colours represent values lower than average.

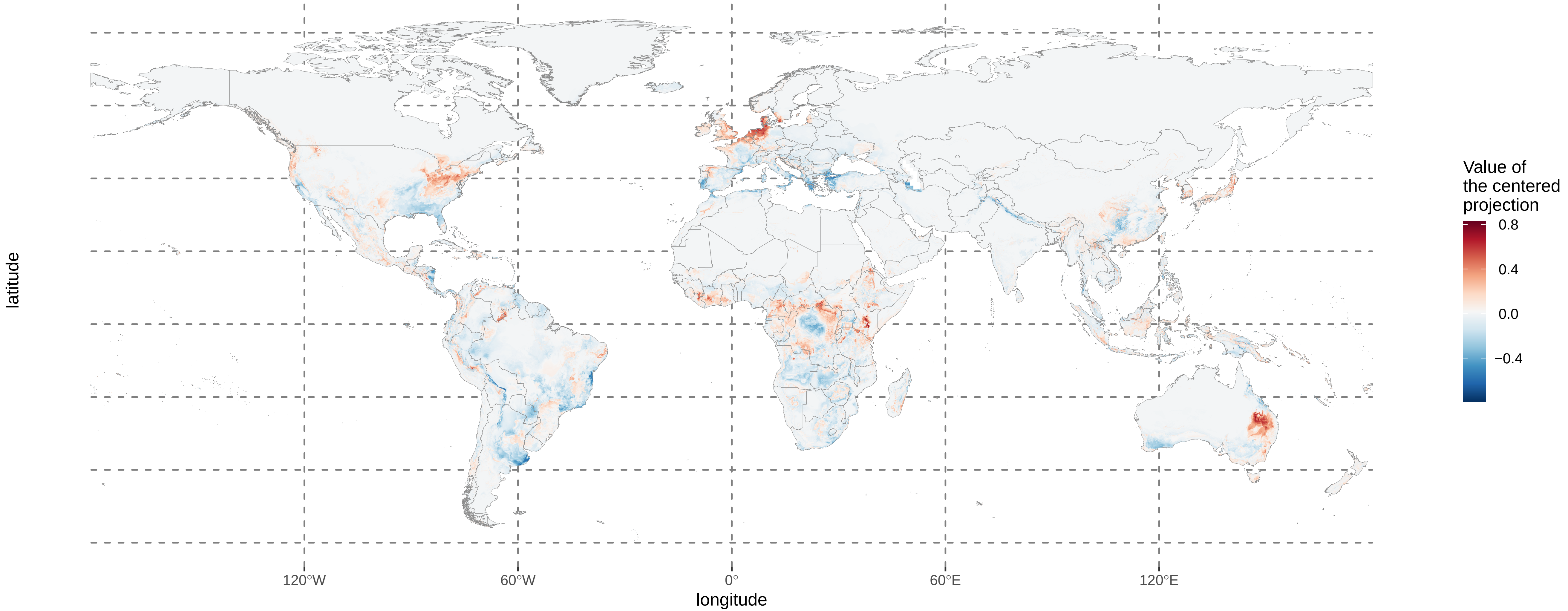

Map illustrating the centered values for the GCM MR in 2070 for the RCP 8.5.
This map was computed by substracting the average value for each pixel to the value for the GCM MR.
Hot colours represent a value higher than average, whereas cold colours represent values lower than average.

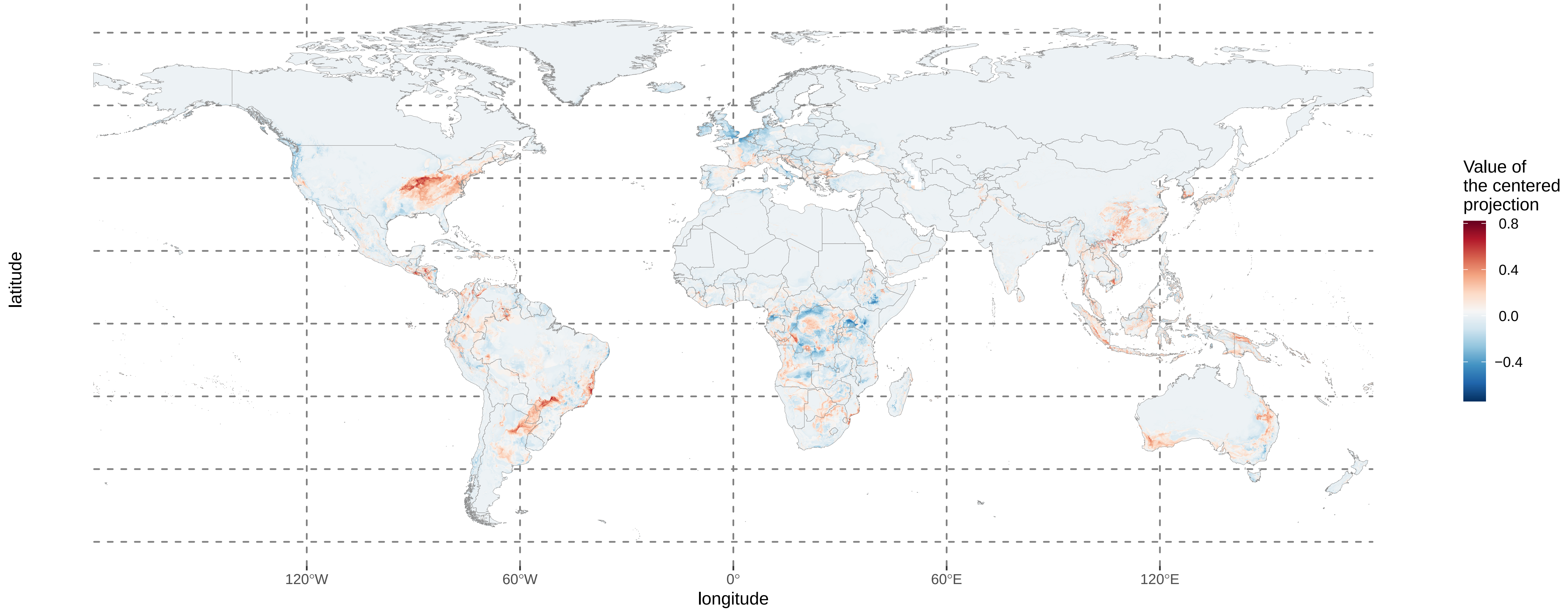

Map illustrating the centered values for the GCM MC in 2070 for the RCP 8.5.
This map was computed by substracting the average value for each pixel to the value for the GCM MC.
Hot colours represent a value higher than average, whereas cold colours represent values lower than average.

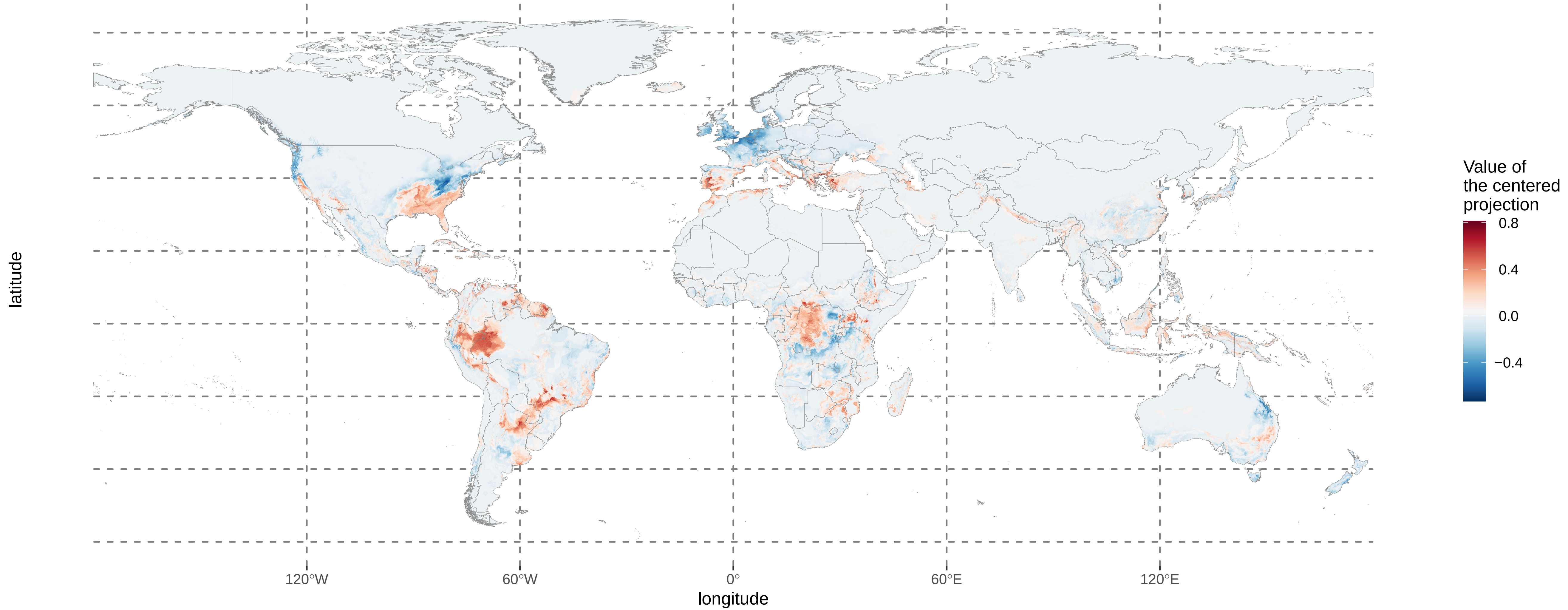

Map illustrating the centered values for the GCM MG in 2070 for the RCP 8.5.
This map was computed by substracting the average value for each pixel to the value for the GCM MG.
Hot colours represent a value higher than average, whereas cold colours represent values lower than average.

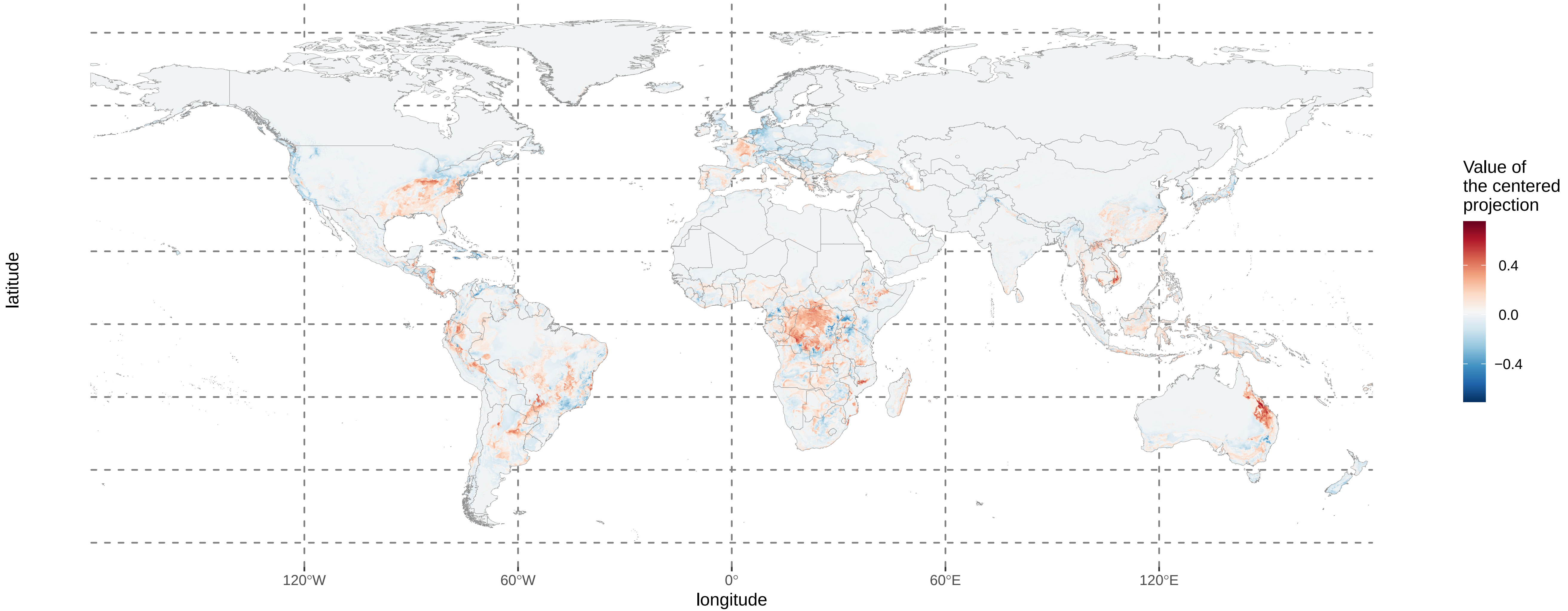

Map illustrating the centered values for the GCM NO in 2070 for the RCP 8.5.
This map was computed by substracting the average value for each pixel to the value for the GCM NO.
Hot colours represent a value higher than average, whereas cold colours represent values lower than average.
